# Trends in worldwide research on cardiac fibrosis over the period 1989–2022: a bibliometric study

**DOI:** 10.3389/fcvm.2023.1182606

**Published:** 2023-06-05

**Authors:** Yukang Mao, Qiangqiang Fu, Feng Su, Wenjia Zhang, Zhong Zhang, Yimeng Zhou, Chuanxi Yang

**Affiliations:** ^1^ Department of Cardiology, the Affiliated Suzhou Hospital of Nanjing Medical University, Suzhou Municipal Hospital, Gusu School, Nanjing Medical University, Suzhou, China; ^2^ Department of Cardiology, The First Affiliated Hospital of Nanjing Medical University, Nanjing, China; ^3^ Department of General Practice, Clinical Research Center for General Practice, Yangpu Hospital, Tongji University School of Medicine, Shanghai, China; ^4^ Department of Cardiology, Yangpu Hospital, Tongji University School of Medicine, Shanghai, China

**Keywords:** cardiac fibrosis, bibliometric, research trend, systematic review, citespace, VOSviewer

## Abstract

**Background:**

Cardiac fibrosis is a hallmark of various end-stage cardiovascular diseases (CVDs) and a potent contributor to adverse cardiovascular events. During the past decades, extensive publications on this topic have emerged worldwide, while a bibliometric analysis of the current status and research trends is still lacking.

**Methods:**

We retrieved relevant 13,446 articles on cardiac fibrosis published between 1989 and 2022 from the Web of Science Core Collection (WoSCC). Bibliometrix was used for science mapping of the literature, while VOSviewer and CiteSpace were applied to visualize co-authorship, co-citation, co-occurrence, and bibliographic coupling networks.

**Results:**

We identified four major research trends: (1) pathophysiological mechanisms; (2) treatment strategies; (3) cardiac fibrosis and related CVDs; (4) early diagnostic methods. The most recent and important research themes such as left ventricular dysfunction, transgenic mice, and matrix metalloproteinase were generated by burst analysis of keywords. The reference with the most citations was a contemporary review summarizing the role of cardiac fibroblasts and fibrogenic molecules in promoting fibrogenesis following myocardial injury. The top 3 most influential countries were the United States, China, and Germany, while the most cited institution was Shanghai Jiao Tong University, followed by Nanjing Medical University and Capital Medical University.

**Conclusions:**

The number and impact of global publications on cardiac fibrosis has expanded rapidly over the past 30 years. These results are in favor of paving the way for future research on the pathogenesis, diagnosis, and treatment of cardiac fibrosis.

## 1. Introduction

Cardiac fibrosis is commonly recognized to be highly related to multiple cardiovascular disorders, such as heart failure (1), atrial arrhythmias (2), and sudden cardiac death (3), whose pathophysiological features can be generalized as excessive extracellular matrix (ECM) production and deposition in the myocardial interstitium. Under most cardiac pathological conditions, the presence and severity of cardiac fibrosis is highly predicative of poor prognosis, while in fact the role of cardiac fibrosis appears more likely to be a double-edged sword. According to different underlying causes and histological characteristics, cardiac fibrosis can be categorized into two major types: reparative fibrosis and reactive interstitial fibrosis (4). Due to the negligible regenerative property of the adult mammalian heart, reparative fibrosis is an indispensable biological process that can replace necrotic cardiac tissues and preserve the geometry and function of the heart to a large extent after injury occurs. A prominent example of reparative fibrosis is myocardial infarction (MI), where fibrotic scar formation maintains the structural integrity of the heart chamber and protects against life-threatening complications such as heart rupture, despite the presence of impaired contractile function (5). In contrast, reactive interstitial fibrosis may occur in the absence of an evident sign of cardiomyocyte loss, leading to increased ventricular stiffness, reduced chamber compliance, diastolic dysfunction, and eventually heart failure with preserved ejection (HFpEF), which is the heart failure phenotype primarily related to metabolic risk factors, including hypertension, diabetes, obesity, and aging (6). In addition, atrial fibrosis has been demonstrated to be closely involved in the pathogenesis of atrial arrhythmias, especially atrial fibrillation (AF) (2). Thus, cardiac fibrosis is a complex and multifaceted response highly depending on the pathophysiological status rather than simply generalized as a single disease entity that would be treated by standardized therapeutic procedures.

According to existing studies, the current knowledge of cardiac fibrosis focuses on three main aspects: (1) an appropriate method for diagnosing cardiac fibrosis is of utmost importance to assess the association between cardiac fibrosis and major clinical outcomes in patients with cardiovascular diseases (CVDs). Endomyocardial biopsy (EMB) is traditionally considered as the “gold standard” for the diagnosis of cardiac fibrosis, whereas such method cannot be routinely utilized due to its inherent disadvantages such as invasive operations, unexpectable complications, and inevitable sampling errors (7). Later, the rapid emergence of non-invasive imaging techniques and sensitive fibrosis biomarkers facilitate the *in vivo* evaluation of cardiac fibrosis. Cardiovascular magnetic resonance (CMR) imaging represents a novel and promising approach for the early detection of cardiac fibrosis, with late gadolinium enhancement (LGE) and extracellular volume (ECV) serving as the clinical references for the assessment of focal and diffuse fibrosis, respectively (8, 9). In addition to radiographic evidence, elevated serological biomarkers, including procollagen type III N-terminal propeptide (PIIINP), procollagen type I C-terminal propeptide (PICP), and galectin-3 (Gal-3) have also been identified as robust predictors of cardiac fibrosis (10, 11); however, lack of cardiac specificity hampers the application of these biomarkers in clinical practice. (2) A comprehensive knowledge of the cellular and molecular mechanisms of cardiac fibrosis is in favor of shedding light on the pathogenesis of the disease. It is generally accepted that the central cellular effectors of cardiac fibrosis are cardiac fibroblasts, whose proliferation, migration, differentiation into cardiac myofibroblasts that express smooth muscle α-actin (α-SMA) as a hallmark, and subsequent production of ECM in the interstitium constitute a chain of events that drive cardiac fibrotic response (4). Besides, alternate cardiac cell types, including cardiomyocytes (12, 13), vascular endothelial cells (14), and cardiac-resident immune cells (e.g., macrophages/monocytes, lymphocytes, and mast cells) (15, 16) may promote cardiac fibroblast activation via releasing multiple profibrotic mediators, thereby accelerating the process of cardiac fibrosis. Molecular mechanisms underlying cardiac fibrosis include neurohormonal activation (e.g., sympathetic nervous system (SNS), renin-angiotensin-aldosterone system (RAAS)) (12, 17), pro-inflammatory cytokines and chemokines [e.g., interleukin-1β (IL-1β), interleukin-6 (IL-6), tumor necrosis factor-α (TNF-α), monocyte chemoattractant protein-1 (MCP-1)] (18, 19), fibrogenic factors [e.g., transforming growth factor-β (TGF-β), endothelin-1 (ET-1), platelet-derived growth factors (PDGFs), connective tissue growth factor (CTGF), Gal-3, exosomal microRNAs (Exo-miRs)] (12–14), and oxidative stress (20). (3) The treatment of cardiac fibrosis has long been an issue of great concern and remains a formidable challenge to clinical practitioners to date. Given little is known about whether established cardiac fibrosis can be effectively attenuated or even reversed, anti-fibrotic therapies centered on interfering with major cellular and molecular components participating in the fibrotic response and risk factors for cardiac fibrosis appear to be more feasible at present. Several drugs [such as RAAS antagonists, sodium-glucose co-transporter-2 (SGLT2) inhibitors, and antioxidants] (21, 22) and lifestyle interventions (23, 24) that are beneficial for cardiovascular fitness have been demonstrated to prevent early cardiac fibrosis in animal models exposed to various cardiovascular risk factors; however, evidence of such beneficial effects in human patients remains uncertain. Ameliorated cardiac fibrosis was found in part of the patients who regularly took medications, experienced left ventricular assist device (LVAD) implantation and artificial valve replacement (25, 26), whereas this improvement was not consistently documented in other patients, especially in those with advanced fibrosis (27, 28). These seemingly paradoxical findings may be attributed to differences in types of fibrotic lesions under various pathological situations and the degree of cardiac fibrosis at the time of being treated. Therefore, further studies are warranted to seek appropriate therapeutic strategies for cardiac fibrosis.

As an undesirable cardiac pathological alteration that accompany most CVDs and a robust contributor to increased cardiovascular morbidity and mortality, cardiac fibrosis has gained increasing attention for decades, particularly on its clinical outcomes, underlying mechanisms, and therapeutic strategies. To date, a large amount of both pre-clinical and clinical studies on cardiac fibrosis have been published; however, a comprehensively visualized bibliometric profile of the literature concerning this issue remains largely unknown. The term “bibliometrics” was a novel concept raised by Alan Pritchard in 1969 and gradually emerged as a separate discipline that predominantly focuses on the influence of publications, contributions of countries/institutions/authors, collaboration between different countries/institutions/authors, and the current hotspots and future trends of a study theme. With the assistance of several sophisticated softwares, bibliometrics can provide the quantitative and qualitative references for assessing the evolving tendencies in a research field of interest over a certain period (29). Herein, we provide a contemporary overview of guidelines for performing a bibliometric study (30). In the present study, we analyzed the bibliometric characteristics of the global research on cardiac fibrosis published over the past three decades to dissect the research status and summarize the current trends and hotspots in this realm.

## 2. Methods

### 2.1. Data sources and search strategy

The bibliometric analysis was performed based on Web of Science Core Collection (WoSSC), which is considered one of the most widely used data sources for bibliometric research and is capable of providing a comprehensive overview of relevant information including publications, citations, authors, references, and keywords (31). The scope of data acquisition was confined to the Science Citation Index-Expanded (SCIE) database, regardless of language. Our search query combined Medical Subject Headings (MeSH) terms and keywords including “myocardial fibrosis” OR “myocardial interstitial fibrosis” OR “cardiac fibrosis” OR “atrial fibrosis” OR “ventricular fibrosis” OR “heart fibrosis” OR “myocard* fibrosis” OR “myocard* interstitial fibrosis” OR “card* fibrosis”. The inclusion criteria were listed as follows: (1) documents published between 1 January, 1989 and 31 December, 2022; (2) document types of “articles”, “reviews”, “editorial material”, and “early access”, thus excluding “meeting abstract”, “proceedings paper”, “book chapter”, and any other non-relevant categories from the results. For the purpose of mitigating confounding bias caused by daily database updates, the retrieval process was implemented on a single day (December 31, 2022). To ensure the authenticity and reliability of the research data, two well-trained investigators independently accomplished the mission of data retrieval, and another colleague was invited to participate in the discussion only in the case where divergent opinions occurred and needed to be resolved. After manual reference screening, we ultimately obtained a total of 13,446 documents, and downloaded full records and cited references in the form of plain text for further analyses. A detailed flow chart of data collection and subsequent analytic procedures were illustrated in Supplementary Figure S1.

### 2.2. Data analysis and visualization

We employed Bibliometrix (version 4.1.3), VOSviewer (version 1.6.17), and CiteSpace (version 5.8.R3) to perform bibliometric analyses, and utilized Microsoft Office Excel 2019 for quantitative analysis of the selected articles. Co-authorship, co-citation, and co-occurrence constitute three important aspects of bibliometric analysis. Co-authorship is defined as the presence of two or more research entities that make equal or hierarchical contributions to a study; therefore, this index can be used to reflect the cooperation between different countries/institutions/authors. Co-citation refers to a situation in which two references or other elements are cited together by subsequent citing papers, with its amount being indicative of the robustness of the relationships between these items (32). Co-occurrence network is typically constructed on the basis of the frequencies of two keywords or other elements appearing in the same documents. Moreover, we reduced the study period to the last 5 years (2016–2022), as well as to the last year (2022), and repeatedly performed the analyses to further detect the evolving trends in research papers published more recently.

Bibliometrix R package, developed by Massimo Aria and Corrado Cuccurullo, is an open-source tool applicable for the generation of a comprehensive science map of the published literature and is freely accessible on Github (https://github.com/massimoaria/bibliometrix) (33). Specifically, Bibliometrix was used in this study to yield a quantitative estimation of annual publication outputs and major journals, and to predict future trends in the research field of cardiac fibrosis.

van Eck et al. introduced a newly developed computer program, namely, VOSviewer (https://www.vosviewer.com), which is capable of visually mapping and displaying bibliometric networks (34). Herein, we applied VOSviewer for implementing bibliographic coupling analysis based on countries, institutions, journals, authors, and references and networks of co-citation, co-authorship, and co-occurrence of keywords, in which each node corresponds to an individual object, with the size of node and the thickness of the line connecting two nodes being representative of the amount or frequency and the strength of the cooperative/co-cited/co-occurring association between different objects, respectively. In addition, clusters share similarities in particular attributes were marked with the same color in the network.

CiteSpace, designed by a Chinese scholar named Chaomei Chen, is a Java-based software tool widely used in visualizing knowledge maps and predicting evolving trends of a research field (35). In this study, we utilized CiteSpace to perform clustering, timeline, and burst analysis of co-cited references and co-occurring keywords and visualize co-citation and co-authorship networks. All cluster labels were extracted from the keywords based on log-likelihood test (*P* < 0.001), and carefully re-checked to determine whether necessary modifications are needed. Timeline view allowed us to explicitly identify the evolution of different research domains. To investigate the properties of each cluster, a series of metrics, including temporal metrics (e.g., citation burst), structural metrics (e.g., betweenness centrality, modularity, silhouette score), and a combined concept of both elements (also known as sigma metrics) was adopted in CiteSpace. Citation burst is a concept corresponding to the circumstance in which a surge of citations of a particular publication occurs during a specific period of time (36). If a cluster incorporates large amounts of nodes with high citation bursts, such cluster may represent an emerging trend in current or future research. Betweenness centrality depends on the frequency a node lies on the shortest pathways between pairs of other nodes (37). If a node was found to possess high betweenness centrality, it was considered a so-called turning point and marked with purple, with the color becoming brighter proportionally with increasing betweenness centrality (35). For instance, papers regarded as turning points typically refer to those experiencing rapid growth in citations within a short period of time or serving as a milestone in the evolution of a specific research domain. The modularity score is also termed as the *Q* score, which is commonly applied for quantifying the extent to which modules or clusters can be obtained from divisions of a network, and whose range of value is from 0 to 1 (38). The cluster structure is considered significant in the case of the *Q* score exceeding 0.3, and possessing a high *Q* score serves frequently as an important feature of a well-structured network. The silhouette score (the *S* score), ranging from −1 to 1, permits the evaluation of the quality of clustering analysis and data configuration (39). A *S* score greater than 0.3, 0.5, and 0.7 is recognized as the major criteria of identifying homogeneity, reasonability, and credibility of a network, respectively. Sigma is an indicator of both structural and temporal properties and is generated through merging betweenness centrality with citation burst [(betweenness centrality + 1)^citation burst^] (40). A higher Sigma value commonly indicates a higher impact. In addition, the symbolic meaning of network structures such as nodes and connecting lines was identical with those in VOSviewer.

## 3. Results and discussion

### 3.1. Analysis of co-cited references: cluster of research and most cited papers

#### 3.1.1. Cluster of research

##### 3.1.1.1. Cluster of research: for 1989–2022 time period

With the use of CiteSpace, we built the cluster-based co-cited reference networks for the period 1989–2022, 2017–2022, and the year 2022, respectively. Each reference was represented by a single node in the network, with its size being positively correlated with the times the reference has been co-cited. The name of the first author with the highest number of citations in each cluster was black-colored and placed adjacent to the cluster label. All these networks presented significant cluster structure and adequate credibility (*Q* = 0.8265, *S* = 0.9308 for the 1989–2022 network; *Q* = 0.7075, *S* = 0.8928 for the 2017–2022 network; and *Q* = 0.6737, *S* = 0.8791 for the 2022 network, respectively). Detailed information about the most important clusters in the network ranked by citation burst were illustrated in Supplementary Figure S2, and descriptions of cluster labels were listed in Supplementary Table S1. In addition, the link walkthrough between clusters based on burst dynamics for co-cited reference network (1980–2022) was shown in Supplementary Figure S3.

**Figure 1:**
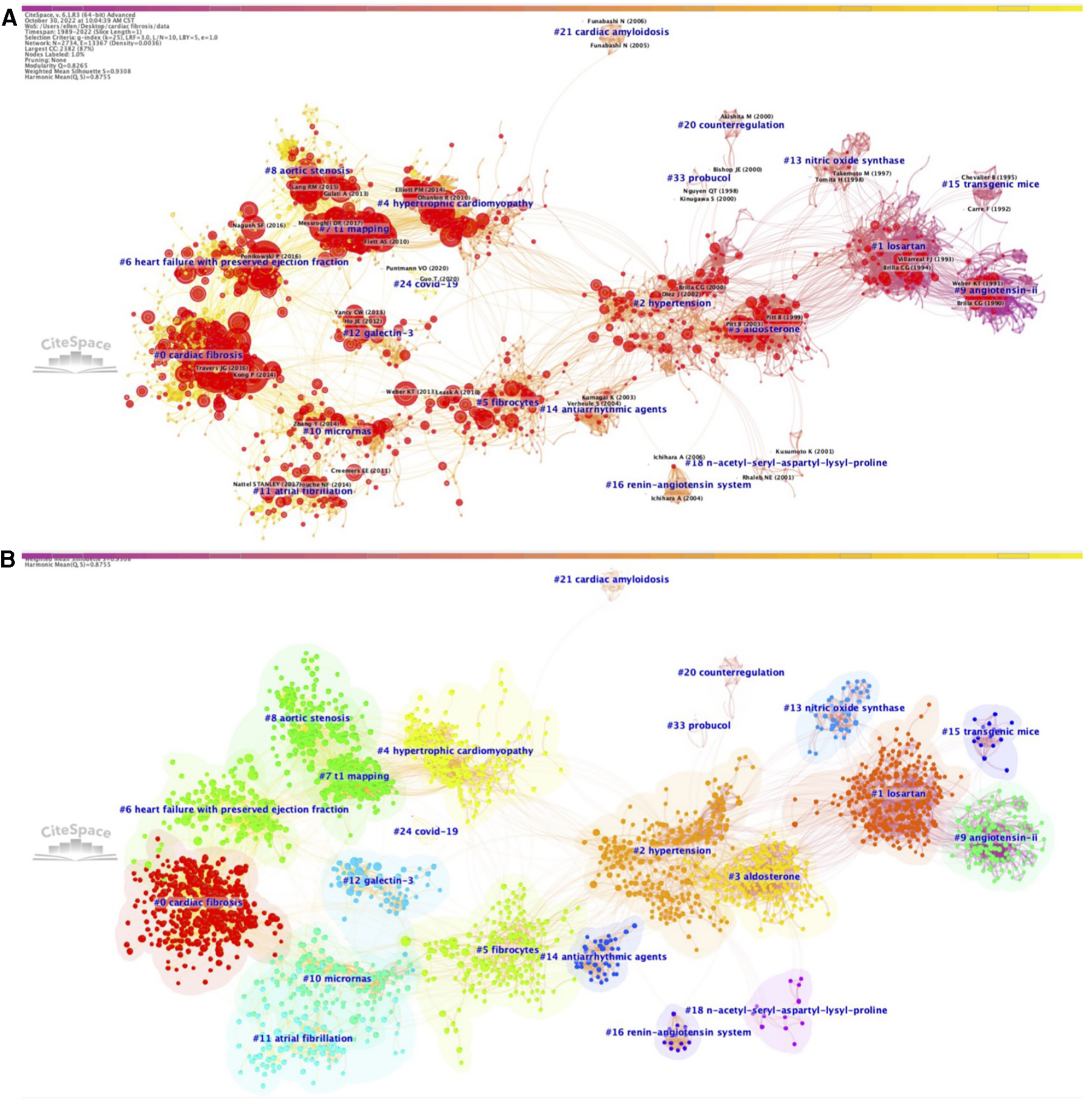
Co-citation references network (1989–2022) and correspondent clustering analysis obtained with citeSpace. (**A**) Co-citation reference network with cluster visualization and burstness of hotspots. (**B**) Visualization map of the corresponding clusters and burestness of hotspots. The size of a node (article) is proportional to the number of times the article has been co-cited. Burstness is represented by red tree rings, with either important citation burst.

Herein, we elaborated on the clusters constituting these trends, with cluster label, cluster number, size (*N*), silhouette score (*S*), mean year (*Y*) of co-cited references, and most representative references, to summarize how research topics in this field developed during the past three decades. As shown in Figure 1, a total of 33 different clusters, which were numbered based on their sizes [ranging from the largest size (#0) to the smallest size (#33)], were identified in the network for the period 1989–2022, and integrated into four major trends of research on cardiac fibrosis, namely, pathophysiological mechanisms (cluster #9, #15, #13, #3, #16, and #5), treatment strategies (cluster #1, #18, #20, #33, #14, and #10), cardiac fibrosis and related CVDs (cluster #2, #21, #4, #8, #11, #6, and #0), early diagnostic methods (cluster #12 and #7), as well as an additional tiny trend on Covid-19 (cluster #24) that emerged more recently.

The first major trend focusing on the pathophysiological mechanisms underlying cardiac fibrosis started around the early 1990s, when researchers observed the presence of marked cardiac fibrosis in transgenic rodent models of arterial hypertension [cluster #9, “transgenic mice” (*N* = 134; *S* = 0.974; *Y* = 1989)] (41), with angiotensin II (AngII) and downregulated expression and activity of nitric oxide synthase (NOS) being the leading causes [cluster #13, “nitric oxide synthase” (*N* = 41; *S* = 0.99; *Y* = 1997)] (42), and that administration of angiotensin converting enzyme inhibitor (ACEI) can effectively attenuate the degree of cardiac fibrosis via suppression of AngII [cluster #15, “angiotensin II” (*N* = 16; *S* = 0.991; *Y* = 1993)] (43). Later, since the early 2000s, this trend extended to cluster #3, “aldosterone” (*N* = 192; *S* = 0.964; *Y* = 2001) (44) that emphasized the role of aldosterone in the pathogenesis of cardiac fibrosis, followed by cluster #16 on “renin-angiotensin system” (*N* = 15; *S* = 1; *Y* = 2006) (45). Another cluster concerning the cellular events that drive cardiac fibrotic response [cluster #5, “fibrocytes” (*N* = 178; *S* = 0.921; *Y* = 2007)] (46) has appeared the last decade and gradually developed into an important study theme since then.

The second major trend focused on treatment strategies for cardiac fibrosis. A cluster on “losartan”, #1 (*N* = 262; *S* = 0.87; *Y* = 1994) (47) emerged as the beginning of this research trend, subsequently evolved into two distinct set of clusters, one of which developed into cluster #18, “n-acetyl-seryl-aspartyl-lysyl-proline” (*N* = 13; *S* = 0.995; *Y* = 2001) (48), and the other one of which incorporated cluster #33, “probucol” (*N* = 4; *S* = 0.999; *Y* = 1998) (49) and cluster #20, “counterregulation” (*N* = 10; *S* = 0.996; *Y* = 1998) (50), and evolved into cluster #14 on “antiarrhythmic agents” (*N* = 39; *S* = 0.995; *Y* = 2004) (51). It is noteworthy that the emergence of a novel cluster on “micrornas”, #10 (*N* = 121; *S* = 0.951; *Y* = 2011) (52) around the 2010s indicated the rapidly increasing interest on the regulation of cardiac fibrosis by microRNAs (miRNAs) and thus may provide revolutionary insights into the treatment strategies for cardiac fibrosis.

The third major trend was centered on cardiac fibrosis itself and related CVDs. This trend of research originated from cluster #2 on “hypertension” (*N* = 226; *S* = 0.899; *Y* = 2002) (53), predominantly due to the high prevalence of hypertension globally and the fact that elevated arterial pressure is one of the most contributors to inducing cardiac fibrosis, while it continued to segregate into two distinct branches, with one branch evolving into a composite of clusters on CVDs associated with ventricular fibrosis, including cluster #4, “hypertrophic cardiomyopathy” (*N* = 179; *S* = 0.922; *Y* = 2008) (54), cluster #21, “cardiac amyloidosis” (*N* = 9; *S* = 1; *Y* = 2005) (55), cluster #8, “aortic stenosis” (*N* = 135; *S* = 0.904; *Y* = 2017) (56), and cluster #6, “heart failure with preserved ejection fraction” (*N* = 146; *S* = 0.94; *Y* = 2016) (57), and the other branch evolving into a cluster on AF, one of the most common CVDs relevant to atrial fibrosis [cluster #11, “atrial fibrillation” (*N* = 94; *S* = 0.959; *Y* = 2013)] (58). With the accumulation of knowledge about different aspects of cardiac fibrosis during the past decades, more on-going studies attempted to conduct experiments or summarize the research domains from a more comprehensive perspective, eventually leading to the formation of cluster #0 on “cardiac fibrosis” (*N* = 350; *S* = 0.926; *Y* = 2016) (59), which is currently the largest cluster.

The fourth major trend, composed of two predominant clusters, occurred based on the need for acquiring an early diagnosis of cardiac fibrosis in clinical practice. Compared to other research trends, formation of this trend was relatively late, starting with cluster #7 on “T1 mapping” (*N* = 145; *S* = 0.928; *Y* = 2013) (60), in which the clinical application of CMR T1 mapping in assisting the diagnosis of cardiac fibrosis were comprehensively assessed. The other independent cluster emerged almost at the same time on “galectin-3”, #12 (*N* = 67; *S* = 0.984; *Y* = 2011) (61), discussing the potential of Gal-3 as a novel biomarker for identifying cardiac fibrosis.

We also found a tiny but unneglectable cluster on “Covid-19”, #24 (*N* = 6; *S* = 0.999; *Y* = 2020) (62). Although the emergence of Covid-19 as a separate cluster appeared to be a serendipity, given cardiac impairment and fibrosis related to Covid-19 started to gain extensive attention from academia since the last 2 years, when Covid-19 pandemic occurred and spread around the world, it might be reasonable that Covid-19 became a novel topic in this field.

##### 3.1.1.2. Cluster of research: for 2017–2022 and 2022 period

In addition, we performed co-citation analyses of references published during the period of 2017–2022 (Supplementary Figures S4, S5) with yearly time slices and the year 2022 with monthly time slices (Supplementary Figure S6), which facilitated us to gain a more comprehensive knowledge of how research trends in this field evolved in more recent years.

The network for the 2017–2022 time period displayed a set of 13 clusters. There was a considerable overlap in identified clusters between this network and 1989–2022 network; however, 4 of these clusters did not occur in the 1980–2022 network: (1) cluster #7 on “EndMT” (*N* = 54; *S* = 0.929; *Y* = 2014) (63) that detected the potential role of endothelial-to-mesenchymal transition (EndMT) in the pathogenesis of cardiac fibrosis; (2) cluster #8 on “relaxin” (*N* = 36; *S* = 0.946; *Y* = 2014) (64) that focused on whether relaxin can be used as a promising candidate drug for the treatment for cardiac fibrosis; (3) cluster #9 on “diabetic cardiomyopathy” (*N* = 29; *S* = 0.966; *Y* = 2017) (65) that paid more attention to cardiac fibrosis in the context of diabetes; (4) cluster #5 on “cardiac magnetic resonance” (*N* = 60; *S* = 0.894; *Y* = 2017) (66) as an extension of the cluster “T1 mapping”. These clusters corresponded to newly emerging directions in different research trends, respectively.

As to the 2022 network, we identified 17 clusters in total and observed the presence of several additional clusters on “myofibroblast”, #0 (*N* = 71; *S* = 0.715; *Y* = 2018) (67), on “leukocyte”, #2 (*N* = 46; *S* = 0.877; *Y* = 2019) (6), on “mitral valve prolapse”, #4 (*N* = 43; *S* = 0.938; *Y* = 2018) (68), on “car-t cells”, #5 (*N* = 35; *S* = 0.935; *Y* = 1990) (69), on “systems biology”, #6 (*N* = 33; *S* = 0.92; *Y* = 2018) (70), on “apoptosis”, #9 (*N* = 24; *S* = 0.878; *Y* = 2019) (71), on “vascular endothelial function”, #10 (*N* = 18; *S* = 0.957; *Y* = 2018) (72), on “mitogen-activated protein kinase kinase kinase 3”, #11 (*N* = 6; *S* = 0.993; *Y* = 2019) (73), on “extracellular matrix”, #12 (*N* = 6; *S* = 0.988; *Y* = 2020) (74), and on “nrg-1”, #17 (*N* = 3; *S* = 1; *Y* = 2019) (75), implying an up-to-date trend towards precise identification of specific cellular components and molecular signals closely involved in the initiation and maintenance of cardiac fibrotic events, which may be favorable to tackling cardiac fibrosis as well as its concomitant complicated situations and creating targeted therapies.

#### 3.1.2. Most cited papers

The top 10 papers with the most citation frequencies published during the period 1989–2022, most of which belonged to reviews, were extracted and illustrated item by item in Table 1. Of all these papers, those ranking within the top 5 positions included a contemporary review of the knowledge about the contribution of activated cardiac fibroblasts, profibrotic mediators, and downstream signaling pathways to fibrotic tissues formation in the state of myocardial injury and potential therapeutic strategies for cardiac fibrosis targeted on cardiac fibroblasts published by Travers et al. in 2016 (365 citations) (76) and the latest European Society of Cardiology (ECS) guideline for the diagnosis and treatment of cardiac fibrosis in patients with different types of heart failure (HF) occurred in the same year (245 citations) (77), followed by Kong et al.'s review discussing the major effectors implicated in the pathogenesis of cardiac fibrosis at cellular and molecular levels (224 citations) (78), Khalil et al.'s research article on dissecting the role of cardiac fibroblast-specific TGF-β-Smad2/3 signaling in pressure overload-induced cardiac fibrosis (215 citations) (79), as well as another high-quality review concerning cell biological processes, molecular mechanisms, and therapeutic opportunities of cardiac fibrosis written by Prof. Nikolaos G Frangogiannis from the Wilf Family Cardiovascular Research Institute, Albert Einstein College of Medicine alone (171 citations) (80), while the rest focused on the current status of CMR and the feasibility of applying this method for early diagnosis of cardiac fibrosis in clinical practice (60, 81–84).

**Table 1:** The top 10 most cited references.

Number of citations in the network	Number of citations in the literature	Cited reference	Year	Source	Vol	Page	Title	Doi	Type of paper	Related cluster in Figure 1
365	811	Travers JG	2016	CIRC RES	118	1021	Cardiac fibrosis the fibroblast awakens	10.1161/CIRCRESAHA.115.306565	Review	0
245	11,918	Ponikowski P	2016	EUR HEART J	37	2129	2016 ESC guidelines for the diagnosis and treatment of acute and chronic heart failure	10.1093/eurheartj/ehw128	Guideline	6
224	734	Kong P	2014	CELL MOL LIFE SCI	71	549	The pathogenesis of cardiac fibrosis	10.1007/s00018-013-1349-6	Review	0
215	423	Khalil H	2017	J CLIN INVEST	127	3770	Fibroblast-specific TGF-beta-Smad2/3 signaling underlies cardiac fibrosis	10.1172/JCI94753	The basic research	0
171	317	Frangogiannis NG	2019	MOL ASPECTS MED	65	70	Cardiac fibrosis: cell biological mechanisms, molecular pathways and therapeutic opportunities	10.1016/j.mam.2018.07.001	Review	0
163	704	Messroghli DR	2017	J CARDIOVASC MAGN R	19	0	Clinical recommendations for cardiovascular magnetic resonance mapping of T1, T2, T2*and extracellular volume: a consensus statement by the Society for Cardiovascular Magnetic Resonance (SCMR) endorsed by the European Association for Cardiovascular Imaging (EACVI)	10.1186/s12968-017-0389-8	Review	7
162	660	Flett AS	2010	CIRCULATION	122	138	Equilibrium contrast cardiovascular magnetic resonance for the measurement of diffuse myocardial fibrosis preliminary validation in humans	10.1161/CIRCULATIONAHA.109.930636	Clinical Trial	7
153	712	Moon JC	2013	J CARDIOVASC MAGN R	15	0	Myocardial T1 mapping and extracellular volume quantification: a Society for Cardiovascular Magnetic Resonance (SCMR) and CMR Working Group of the European Society of Cardiology consensus statement	10.1186/1532-429X-15-92	Review	7
145	7,011	Lang RM	2015	EUR HEART J-CARD IMG	16	233	Recommendations for cardiac chamber quantification by echocardiography in adults: an update from the American Society of Echocardiography and the European Association of Cardiovascular Imaging	10.1093/ehjci/jev014	Guideline	8
143	648	Mewton N	2011	J AM COLL CARDIOL	57	891	Assessment of myocardial fibrosis with cardiovascular magnetic resonance	10.1016/j.jacc.2010.11.013	Review	7

We also performed the burst analysis to explore the citation bursts of references for the period 1989–2022 and 2017–2021, respectively (Supplementary Tables S2S–V). The blue line is the timeline sliced year by year, and the red line is representative of how long a citation burst persists. The results implied that the top 3 references with the latest and strongest beginning of citation bursts were “Fibroblast-specific TGF-β-Smad2/3 signaling underlies cardiac fibrosis” published by Khalil et al. in 2017 (79), “Myocardial Interstitial Fibrosis in Heart Failure: Biological and Translational Perspectives” published by González et al. in 2018 (1), and “Cardiac fibrosis: Cell biological mechanisms, molecular pathways and therapeutic opportunities” published by Nikolaos G Frangogiannis (80) in 2019. As for the last 5 years, the top 3 references were a randomized controlled trial (RCT) conducted by Zinman and colleagues for the purpose of evaluating the effects of a sodium-glucose cotransporter 2 (SGLT2) inhibitor on adverse cardiovascular outcomes in patients with type 2 diabetes (85), Lang et al.'s article presenting the updated recommendations for the quantification of the size and function of cardiac chambers using echocardiography from the American Society of Echocardiography/European Association of Cardiovascular Imaging (ASE/ESCVI) (83), and a review created by Nikolaos G Frangogiannis as mentioned above (80).

### 3.2. Analysis of co-occurrence of keywords

**Figure 2:**
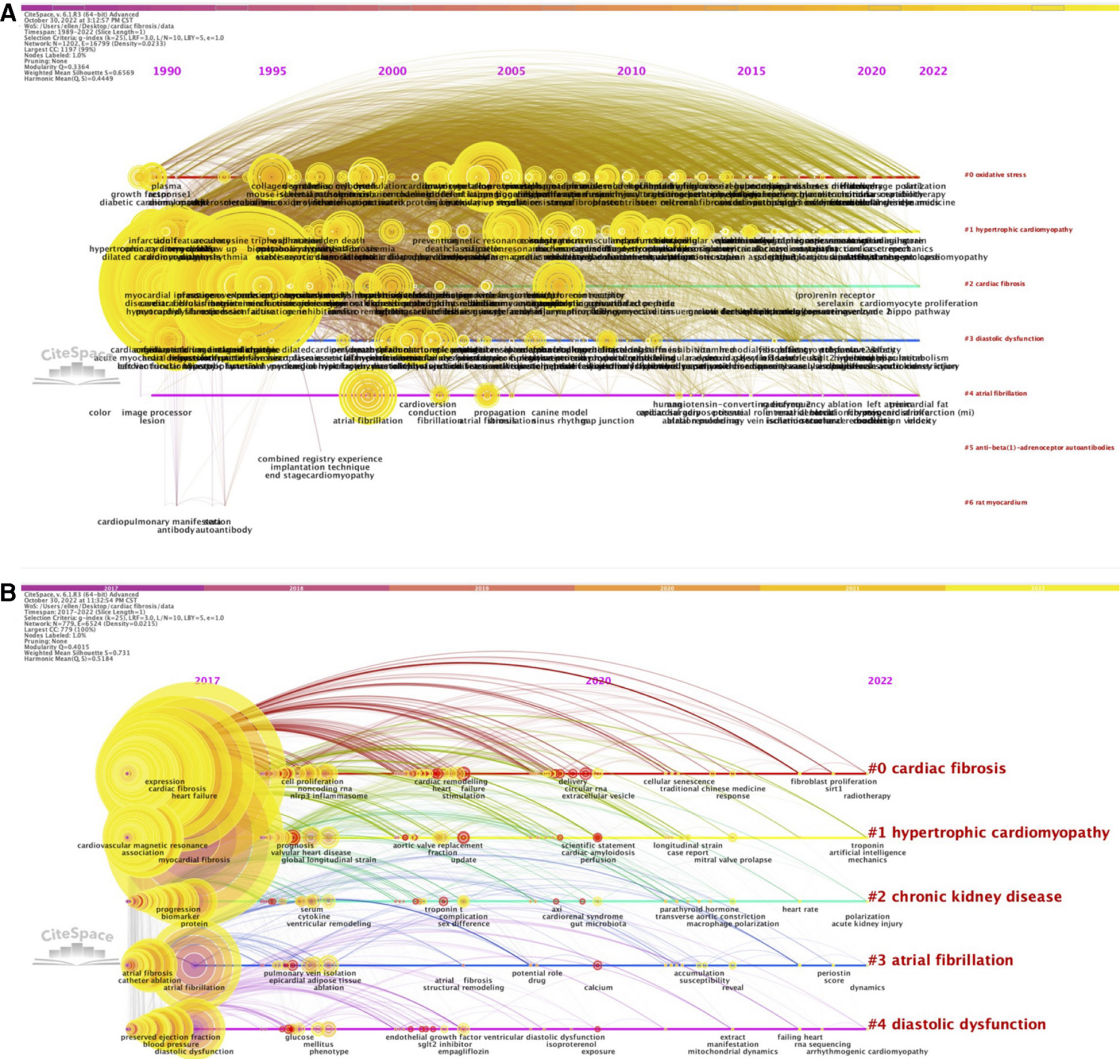
Timeline visualization of co-occurring author keywords networks [(**A**) 1989–2022 and (**B**) 2017–2022]. The nodes represent keywords, and the colors show the average year of publication for each node. The size of tree ring is proportional to the burstness of keyword co-occurrence. The co-occurrence network is weighted on total link strength across different keyword nodes and scored on the average publication years. The clusters are labeled in red at the far right of the timeline maps.

The primary purpose of identifying co-occurring keywords is to provide a comprehensive summary of the major trends in a specific research field and predict the evolution of research hotspots over time. Each node in the co-occurrence network represents a highly co-occurring keyword, with the size of node depending on how frequently a keyword occur. Figure 2 showed the co-occurrence networks of keywords for the period 1989–2022 and 2017–2022, both of which possessed significant modularity and silhouette scores (*Q* = 0.3364, *S* = 0.6569 for the 1989–2022 network; and *Q* = 0.4015, *S* = 0.731 for the 2017–2022 network, respectively).

For the 1989–2022 network, seven distinct clusters were found: cluster #0, “oxidative stress”, cluster #1, “hypertrophic cardiomyopathy”, cluster #2, “cardiac fibrosis”, cluster #3, “diastolic dysfunction”, cluster #4, “atrial fibrillation”, cluster #5, “anti-beta(1)-adrenoceptor autoantibodies”, and cluster #6, “rat myocardium”, while five different clusters were presented in the 2017–2022 network: cluster #0, “cardiac fibrosis”, cluster #1, “hypertrophic cardiomyopathy”, cluster #2, “chronic kidney disease”, cluster #3, “atrial fibrillation”, and cluster #4, “diastolic dysfunction”.

Keywords with high citation bursts can serve as potent predictors of future directions which major research trends and frontiers evolve towards. We observed that the top 3 keywords with the most recent emergence and strongest beginning of citation bursts included “left ventricular dysfunction”, “transgenic mice”, and “matrix metalloproteinase” for the 1989–2022 network, while those for the 2017–2022 network were “cardiorenal syndrome”, “perfusion”, and “reactive oxygen specy” (Supplementary Tables S2W–Z).

We also utilized VOSviewer to construct a network visualization of keyword analysis, in which the thickness of the connecting lines between pairs of keywords is proportional to the frequencies of their co-occurrence, and an overlay visualization of keyword analysis, in which the color of one keyword varies from blue to yellow according to the average year of publication of all the articles containing this keyword (blue color for the keywords occurring in earlier years, and yellow color for those appearing later). From these visualized maps of co-occurring keywords, we screened out five separate clusters marked with different colors, all of which shared considerable similarities in the development of research trends, and found that there was a nearly even distribution of newly emerging topics across these clusters (Supplementary Figure S7).

### 3.3. Publication outputs and major journals

As shown in Supplementary Figure S1, we originally retrieved 15,762 articles on cardiac fibrosis from the WoSSC database, while ultimately included 13,446 articles for bibliometric analysis following strict data filtration. The annual amounts of publications showed a pattern of exponential growth from 1989 to 2022, including a dramatic increase during the last 5 years (2017–2022), and reached its peak in 2021, indicating that cardiac fibrosis is a prevalent research topic during the past three decades and is likely to continue to thrive. An increasing trend was also observed in the average number of citations per year during the same period, with the highest citations appearing in 2019; however, the increasing course appeared to be slower and more tortuous (Supplementary Figure S8).

We extracted 10 journals with the most publications on cardiac fibrosis and the growth trends of the cumulative numbers of publications over time in these journals in Supplementary Figure S9. For the 1989–2022 time period, the top 10 journals were Plos One, Circulation, Cardiovascular Research, Hypertension, American Journal of Physiology-Heart and Circulatory Physiology, International Journal of Cardiology, Journal of Molecular and Cellular Cardiology, Journal of The American College of Cardiology, Scientific Reports, and Frontiers in Cardiovascular Medicine, and we found a stable growth in the cumulative number of publications in most journals, expect Frontiers in Cardiovascular Medicine, Journal of Molecular and Cellular Cardiology, Plos One, and Scientific Reports, in which total amounts of publications were zero or at a low level before 2010 and began to explosively increase since then. It is noteworthy that, from 2017 to 2022, there was an approximately linear growth in the number of publications in most of the top 10 journals, wherein Frontiers in Cardiovascular Medicine is the only one that experienced a turning point during this period. The rising speed of number of publications on the right side of the turning point was obviously higher than that on the left side of the turning point, leading to a surge of publications since 2020 and ultimately enabling the cumulative numbers of publications in Frontiers in Cardiovascular Medicine to exceed those of the other journals. We also provided an overlay visualized map of the most cited journals over the last 5 years with CiteSpace, and built the networks of co-cited journals for the past 30 years with VOSviewer (Supplementary Figure 10).

In addition, for the period 1989–2022, we detected the latest and strongest citation burst in International Journal of Molecular Sciences (170.87), followed by Frontiers in Physiology (111.62) and Cells-Basel (108.64) (Supplementary Tables S2G,H), while for the period 2017–2022, the latest and strongest citation burst was found in Acta Biochimica Polonica (7.75), followed by European Journal of Human Genetics (7.75) and Frontiers in Microbiology (6.09) (Supplementary Tables S2I,J).

### 3.4. Analysis of cooperation network across countries and institutions

**Figure 3:**
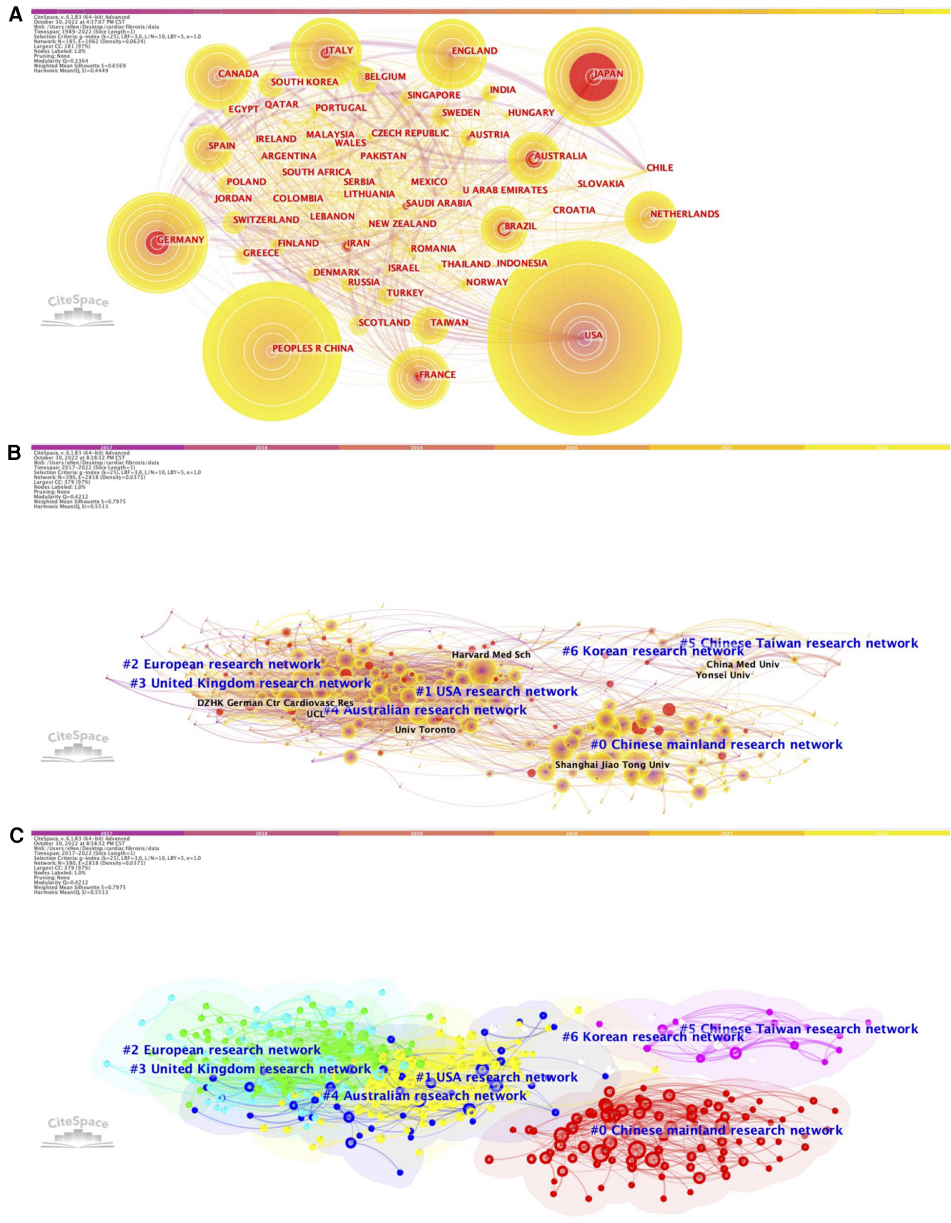
Network of the co-authors’ countries (1989–2022, **A**) and the network of co-authors institutions (2017–2022, **B**) (2017–2022, **C**) for cardiac fibrosis obtained with citeSpace.

We constructed the co-citation networks of countries and institutions (Figure 3), and listed the top countries and institutions ranked by number of citations and betweenness centrality (Supplementary Table S5). Among all the countries or regions that contributed to relevant articles in this field, publications generated by the United States possessed both the highest citation frequencies (*n* = 3,814, 28.37%) and betweenness centrality (0.59) over the period 1989–2022. China ranked second in total citation counts (*n* = 3,544, 26.36%), followed by Germany (*n* = 1,146, 8.52%), Japan (*n* = 1,031, 7.68%), and England (*n* = 773, 5.75%), whereas France ranked second in betweenness centrality (0.27), followed by Germany (0.25), Italy (0.2), and Japan (0.17). On the other hand, over the last 5 years (2017–2022), the United States retained a leading position in the rankings of betweenness centrality (0.27), exceeding England (0.18), France (0.16), Canada (0.13), Germany (0.11), and any other countries/regions; however, China became the area with the highest number of global citations during this period (*n* = 2,592), followed by the United States (*n* = 1,777), Germany (*n* = 516), England (*n* = 462), and Italy (*n* = 373).

When focusing on the most cited institutions, the majority of the top 10 influential institutions were universities from China, especially those highlighting the importance of medical education and advancing medical research. For the period 1989–2022 and 2017–2022, total citations count of publications from Shanghai Jiao Tong University (*n* = 185 and *n* = 180, respectively) and Nanjing Medical University (*n* = 145 and *n* = 143, respectively) ranked first and second. Capital Medical University ranked third (*n* = 165), followed by Monash University (*n* = 162) and Wuhan University (*n* = 149) between 1989 and 2022, while Harvard Medical School ranked third (*n* = 135), followed by Wuhan University (*n* = 113) and Fudan University (*n* = 106) between 2017 and 2022. In terms of betweenness centrality, institutions in the United States and Europe performed the best, with the top position belonging to Harvard University (0.06) for the period 1989–2022 and University of Pittsburg (0.09) for the period 2017–2022, respectively.

According to the results of burst analysis, the latest top 3 countries which possessed the highest citation bursts over the period 1989–2022 included Iran (7.66), Saudi Arabia (5.22), and Indonesia (4.07) (Supplementary Tables S2A,B). As for the institutions, the most recent and strongest citation burst was found in Central South University (19.25), followed by Chinese Academy of Medical Sciences & Peking Union Medical Collage (16.08) and Sichuan University (11.77) for the period 1989–2022 (Supplementary Tables S2C,D), and was found in Central South University (7.64), followed by Emory University (4.78) and Chinese Academy of Medical Sciences & Peking Union Medical Collage (4.29) for the period 2017–2022 (Supplementary Tables S2E,F).

### 3.5. Analysis of co-authorship network

**Figure 4:**
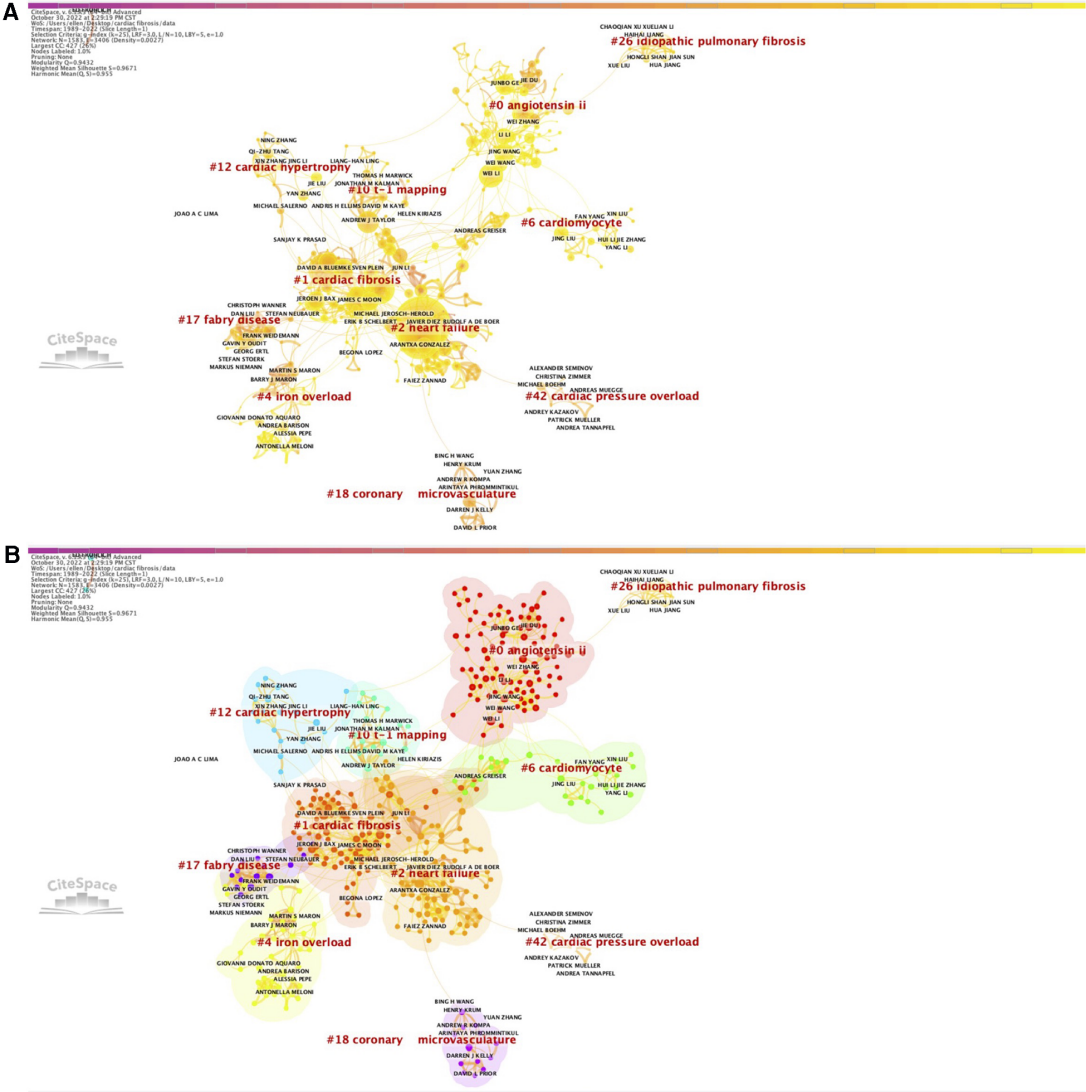
Co-authorship network (**A**) with corresponding clusters (**B**) from 1989 to 2022.

The co-authorship network based on the number of co-authored papers were generated by CiteSpace to visualize the collaboration between different researchers, which can be further extended to the collaboration between their affiliated countries and institutions, and assess the relatedness between their research domains. As shown in Figure 4, the co-authorship network exhibited significant modularity and cluster structure (*Q* = 0.9432, *S* = 0.9671), and suggested that the most recent and most important clusters were cluster #0, “angiotensin II”, cluster #1, “cardiac fibrosis”, and cluster #2, “heart failure” (Supplementary Table S3). We also used VOSviewer to produce a similar co-authorship network, in which a total of 625 authors were presented as single nodes with different sizes and colors and clustered into 51 clusters (Supplementary Figure 1^1^). According to the findings of the burst analysis, the co-authors that were the most frequently involved in the articles published during the period from 1989 to 2022 were Weber KT, Brilla CG, and Bluemke DA (Supplementary Tables S2K,L), and that those most frequently participating in the articles published during the period 2017–2022 were Bluemke DA, Packer M, and Tang QZ (Supplementary Tables S2M,N).

We further constructed the co-citation network that allowed us to obtain a visualized knowledge of the relation of citing/being cited among the authors for the last 5 years (Supplementary Figure 1^2^). The latest top 3 co-cited authors with the strongest strength of citation bursts were Travers JG, Ponikowski P, and Khalil H for the period 1989–2022 (Supplementary Tables S2O,P), and were Small EM, Qu XF, and Suthahar N for the period 2017–2022 (Supplementary Tables S2Q,R).

### 3.6. Bibliographic coupling analysis of countries, institutions, journals, references, and authors

**Figure 5:**
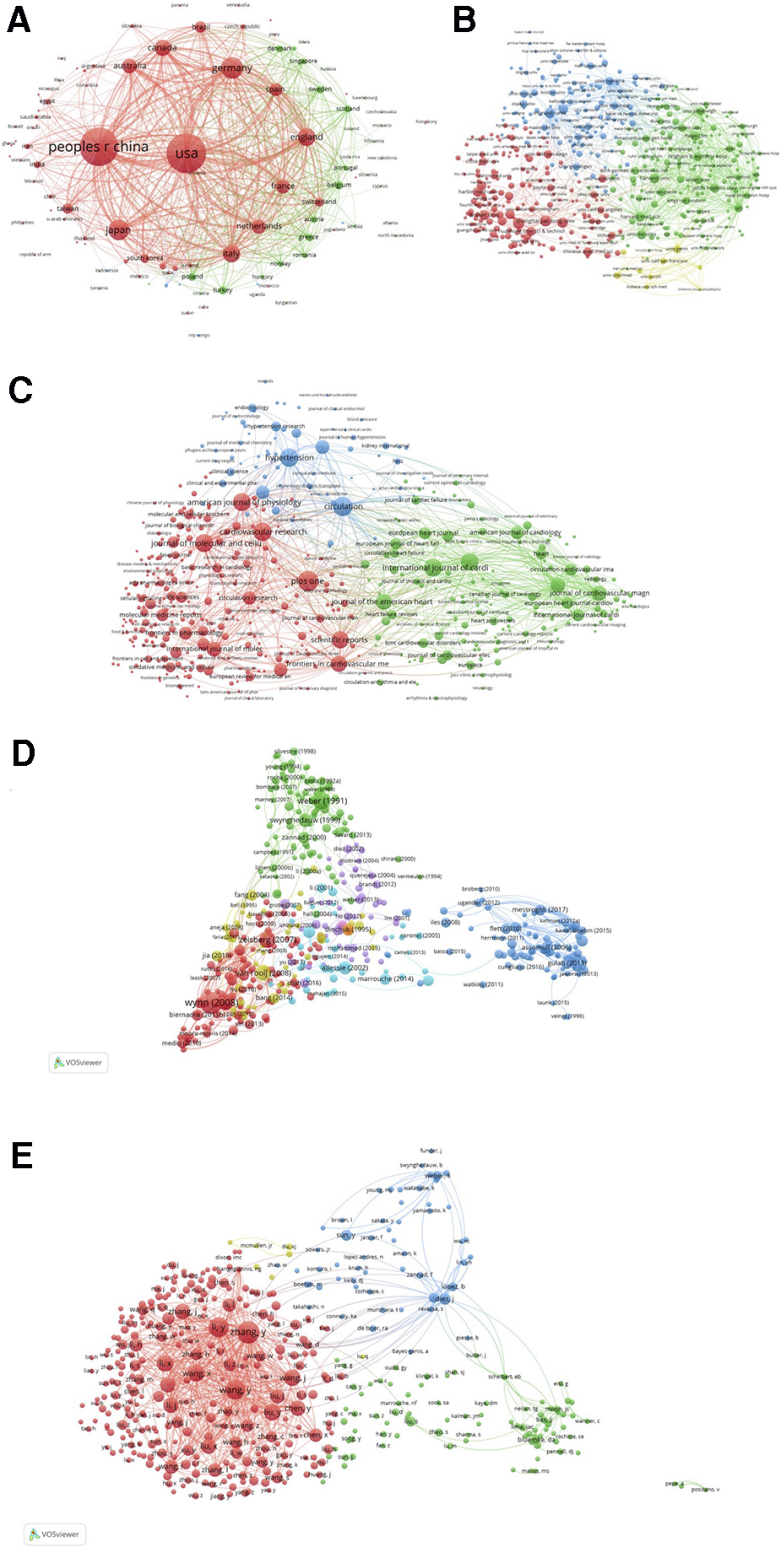
Map of bibliographic coupling analysis based on countries (**A**), institutions (**B**), journals (**C**), references (**D**), and authors (**E**) (weights on the total link strength). Minimum number of documents of a country = 1, 108 meet the thresholds; Minimum number of citations of a document = 150, 455 meet the thresholds; Minimum number of documents of a journal = 5, 475 meet the threshold; Minimum number of documents of an author = 20, 414 meet the threshold; Minimum number of documents of an institution = 15, 459 meet the thresholds.

Since Kessler pioneered a theory that scientific papers sharing similarities in references citation are likely to be topically related to each other in 1963, the concept of bibliographic coupling is thus formed, and is commonly considered an index that can reflect the relatedness between previous publications (86). Herein, VOSviewer was employed to analyze the bibliographic coupling of the publications (the number of publications of a country should exceed 1,108) produced by 129 countries (Figure 5A). The top 5 countries with largest total link strength were the United States (total link strength equals to 4,295,363 times), Sweden (total link strength equals to 2,654,429 times), China (total link strength equals to 2,632,851 times), Germany (total link strength equals to 1,515,547 times), and England (total link strength equals to 1,412,788 times) (Supplementary Table S6).

The findings of the bibliographic coupling of the publications (the number of publications of an institution should exceed 15,459) originating from 459 institutions revealed that the top 5 institutions with largest total link strength were UCL (total link strength equals to 342,731 times), University of Navarra (total link strength equals to 318,797 times), University of Pittsburgh (total link strength equals to 312,393 times), Harvard Medical School (total link strength equals to 311,148 times), and Monash University (total link strength equals to 295,605 times) (Figure 5B; Supplementary Table S6).

The bibliographic coupling network of the publications (the number of publications of a journal should exceed 5,475) published in 475 journals was illustrated in Figure 5C. In this network, we identified the top 5 journals that possessed largest total link strength, including Journal of the American College of Cardiology (total link strength equals to 217,247 times), Journal of Molecular and Cellular Cardiology (total link strength equals to 212,831 times), Cardiovascular Research (total link strength equals to 206,321 times), Circulation (total link strength equals to 199,152 times), and JACC-Cardiovascular Imaging (total link strength equals to 188,831 times) (Supplementary Table S6).

We also detected the bibliographic coupling of the publications (the number of citations of a publication should exceed 150,455) that cited 455 common references (Figure 5D). The top 5 references with largest total link strength were “Cardiac fibrosis: Cell biological mechanisms, molecular pathways and therapeutic opportunities” published by Frangogiannis, N. G. in 2019 (total link strength equals to 1,271 times), “The pathogenesis of cardiac fibrosis” published by Kong et al. in 2014 (total link strength equals to 1,035 times), “Cardiac Fibrosis: The Fibroblast Awakens” published by Travers et al. in 2016 (total link strength equals to 790 times), “Molecular mechanisms of myocardial remodeling” published by Swynghedauw, B in 1999 (total link strength equals to 674 times), and “Assessment of myocardial fibrosis with cardiovascular magnetic resonance” published by Mewton et al. in 2011 (total link strength equals to 617 times) (Supplementary Table S6).

Finally, the bibliographic coupling analysis of publications (the number of documents of an author should exceed 20,414) belonging to 414 authors were performed. As shown in Figure 5E, the top 5 authors with largest total link strength were as follows: Zhang Y (total link strength equals to 564,914 times), Li Y (total link strength equals to 459,824 times), Wang Y (total link strength equals to 452,174 times), Li X (total link strength equals to 330,875 times), and Wang X (total link strength equals to 328,994 times) (Supplementary Table S6).

## 4. Discussion

### 4.1. Summary of the main findings

We provided, for the first time, a detailed and comprehensive overview of global publications on cardiac fibrosis over the past 30 years, predominantly focusing on current research status and the development of major trends and future hotspots. We confirmed that the yearly number of articles on cardiac fibrosis conforms to exponential growth until the year 2021 and is promising to maintain a rising trend in the future. The United States was the most cooperative country and had the most prominent impact in this research field for the period 1989–2022; however, for the last 5 years, China became a brand-new and robust source of global citations, with its total citation count surpassing that of the United States during this period, despite the fact that China lacks adequate collaboration with other countries. Shanghai Jiao Tong University remained the institution with the most citations throughout the entire period from 1989 to 2022, while Harvard University and University of Pittsburg played the most crucial roles in the international cooperative network within the last three decades and within the last 5 years, respectively. The most recently emerging and highest citation burst was found in International Journal of Molecular Sciences, followed by Frontiers in Physiology and Cells-Basel. As for the most influential authors, the top 3 authors with the latest and strongest strength of citation bursts were Travers JG, Ponikowski P, and Khalil H. We also detected the similarities in the research topics via analyzing bibliographic coupling among the articles derived from different countries, institutions, journals, and authors.

### 4.2. Identification of research trends

The co-cited reference network (1989–2022) divided all the included documents into 33 different clusters, which was representative of several major research trends as we mentioned above. The first research trend focuses on pathophysiological mechanisms of cardiac fibrosis. The beginning of this trend was based on experimental observations that transgenic rodent models with hypertension, such as spontaneously hypertensive rats (SHR) tend to exhibit varying degrees of cardiac fibrosis (41), and further evolved into dissection of the role of major components of RAS (e.g., AngII, aldosterone) (87, 88) and suppressed endothelium-derived nitric oxide (NO) synthesis (89) in the pathogenesis of cardiac fibrosis. More recently, this trend converted into an overwhelmingly increasing focus on the contribution of cardiac fibroblast as the most important ECM-producing cell types in promoting cardiac fibrosis (76).

Next, treatment strategies for cardiac fibrosis constituted another major trend, starting with a focus on losartan, a commonly used AngII receptor type 1 (AT1R) blocker (ARB), and its actions of blunting the progression of cardiac fibrosis, demonstrating the crucial role of RAS in driving cardiac fibrotic response from another perspective (90). Subsequently, this trend evolved towards the goal of seeking more drugs based on molecules with newly identified connections with RAS, either by N-acetyl-seryl-aspartyl-lysyl-proline (Ac-SDKP), which is an endogenous polypeptide with potent antifibrotic properties and mediates ACEI-induced reversal of cardiac fibrosis to a certain extent (91), or through counterregulatory effects of AngII receptor type 2 (AT2R) on attenuating cardiac fibrosis caused by AT1R activation (50). A cholesterol-lowering agent, namely, probucol was noted to prevent the development of cardiac fibrosis almost during the same period, probably owing to its antioxidant capacities (49). In addition to pharmacological interventions targeted on preserving the normal structure of the fibrotic heart, some antiarrhythmic agents, such as β-adrenergic blockers, were also considered to improve cardiac fibrosis as well as reduce the prevalence of arrhythmias due to differences in electromechanical characteristics between fibrotic tissue and normal myocardium, especially AF and life-threatening ventricular tachycardia (92). The latest topic was centered on miRNAs as a class of novel regulators of the process of fibrogenesis and thereby a promising therapeutic target for treating cardiac fibrosis (52).

The third trend of research concerned cardiac fibrosis itself and related CVDs, including not only CVDs in favor of the generation of cardiac fibrosis, such as hypertension, hypertrophic cardiomyopathy (HCM), and aortic stenosis (AS), but also those in which cardiac fibrosis is found to be typical pathological manifestations or serve as one of the leading causes, such as HFpEF, cardiac amyloidosis, and AF. In terms of the time order of emergence, hypertension was the earliest cardiac fibrosis-related CVD noticed by researchers (53). Later, the other clusters of research were divided into two major parts, one of which was composed of CVDs characterized by ventricular fibrosis (including HCM, AS, cardiac amyloidosis, and HFpEF) (6, 28, 93, 94), and the other one of which focused on CVDs related to atrial fibrosis, AF in particular (51). These studies were ultimately integrated into an independent research direction in which each aspect of cardiac fibrosis can be systematically and comprehensively explored as a whole (59).

Finally, the rapid development of advanced imaging technique and highly sensitive serological biomarkers gives rise to the later emergence of a trend on early diagnostic methods for cardiac fibrosis. CMR T1 mapping has been generally accepted as a commonly used non-invasive method applicable for conveniently and efficiently assessing the severity of cardiac fibrosis (60), while a growing body of evidence also indicated that Gal-3, a newly identified member of the β-galactoside-binding lectin family, is a promising circulating biomarkers for identifying individuals with high risk of cardiac fibrosis and forecasting their prognosis (95).

In addition, a separate cluster of research has highlighted the importance of investigating the relatedness between cardiac fibrosis and Covid-19. Since the outbreak of Covid-19 pandemic in 2020, articles reporting that cardiac fibrosis is a highly prevalent cardiovascular outcome of Covid-19 infection, preceded by inflammatory cytokines storm and myocardial injury, has gradually increased worldwide, and thus formed a novel trend (62).

To consolidate the findings obtained from clustering analysis, the citation burst of keywords can also favor the identification of the most recent research trends, with the highest citation bursts appearing in similar topics (Supplementary Tables S2W–Z). In addition, articles with the highest citation counts or strongest citation bursts in recent years were predominantly reviews and clinical guidelines, which reflects the abundant accumulation of evidence extracted from numerous experimental discoveries and clinical practice in this field (Table 1; Supplementary Tables S2S–V). Although the past three decades have witnessed the remarkable advancements in research on cardiac fibrosis worldwide, it is noteworthy that there still exist two gaps of knowledge: (1) the wide application of single cell RNA-sequencing (scRNA-seq) and spatial transcriptome technologies permits a novel dimension of examining the alternations in distribution and abundance of specific genes in an individual cell or an anatomical region of interest, however, evidence on the molecular profiles of each cellular component constituting cardiac tissues in the state of cardiac fibrosis is still inadequate. In other words, it is necessary to yield a more profound insight of the origin of cardiac fibrosis at genetic and epigenetic levels and perform *in vitro* and *in vivo* experiments to confirm the function of genes specific to major cell populations involved in the formation of cardiac fibrosis via conditional gene editing tools such as Cre-loxp or CRISPR-Cas9 system; (2) although transgenic or drug-induced rodent models of cardiac fibrosis are indispensable tools for exploring its fundamental mechanisms, these animal models cannot recapitulate the pathophysiologic complexity of cardiac fibrotic response in human patients, which is the reason why we should attach adequate importance on constructing human-based models or conducting more clinical research to overcome the translational barriers and to develop innovative therapeutic strategies.

### 4.3. Strengths and limitations

As far as we know, this study provides an up-to-date summary of the bibliometric characteristics of global publications on cardiac fibrosis during the past 30 years, which may be favorable for medical researchers and clinicians to gain a comprehensive knowledge of the current research status in this research field. The most prominent advantage of our study is the application of various visualization softwares, including Bibliometrix, VOSviewer, and CiteSpace, for the graphical presentation and interpretation of major bibliometric indexes (co-citation, co-occurrence, co-authorship, and bibliographic coupling), which facilitates the identification of evolving research trends, frontiers, and hotspots over time, thus paving the way for advancing future medical research and formulating appropriate therapeutic strategies to prevent such cardiac pathological condition. This novel mode of research allows us to explore the development of a research domain of interest during a certain period of time in a more unbiased manner, compared to those kinds of “old-fashioned” narrative reviews that are prone to be affected by man-made selection bias.

However, we have to acknowledge that our study has several shortcomings that need to be noted and addressed in further research: (1) WoSSC database was chosen as the data source in our study; however, there still exist some other online databases in which publication information are not presented in similar patterns that can be used for bibliometric research, such as Pubmed, Embase, Scopus, and Google Scholar, posing a formidable challenge for merging references published in different databases. In other words, we may not completely guarantee the neutrality of data collection due to variations between different databases; (2) Since the document type included in our study was merely restricted to original article and review (including early access), we cannot fully elucidate the entire features of all publications contributing to this field. Thus, caution should be taken during the analysis and interpretation of the data; (3) Under normal circumstances, the relation of citing or being cited should be determined in an unprejudiced manner for promoting the extension of the territories of knowledge on the basis of previously established evidence; however, it may sometimes be distorted by far-fetched or improper citations of non-relevant literature, which is another potentially existing bias when an author attempts to cite an article to support his/her own opinions and may lead to the situation in which statements without solid foundations are mistakenly recognized as truth by careless readers (96). (4) Given first author was the only focused element in the co-citation networks, how to quantify the impacts of the other co-authors remains to be resolved by future research.

## 5. Conclusion

This study provides a comprehensive insight into contemporary bibliometric characteristics of global publications on cardiac fibrosis throughout the last 30 years (1989–2022), with specific attention on the evolution of research trends. The number of publications has increased exponentially during this period, reaching its peak in 2021. We identified the most influential countries, and authors, and found several major trends of research, pathophysiological mechanisms, treatment strategies, cardiac fibrosis and related CVDs, early diagnostic methods, as well as Covid-19. China is a rapidly emerging source of publication impacts, whereas more cooperation is required between institutions from China and those from the United States and Europe. Our study provides important references for clinical practitioners, medical researchers, and policymakers to obtain a better knowledge of the evolving trends, frontiers, and hot topics of global research on cardiac fibrosis.

## Data Availability

The original contributions presented in the study are included in the article/**Supplementary Material**, further inquiries can be directed to the corresponding authors.

## Supplementary material

Supplementary Figure S1 Flow chart of the scientometric study.

Supplementary Figure S2 Detail focus on most important clusters of the co-citation reference networks ranked by burstness of citations (1989-2022). For each cluster, we report all five top keywords obtained, the selected label being the keywords that are the most cited (generated by the likelihood ratio of keywords). These keywords are highly susceptible to represent the overall topic of a cluster. Burstness is represented in each cluster with red tree-rings around nodes.

Supplementary Figure S3 Link walkthrough between clusters based on burstness dynamic for co-cited reference network (1989-2022).

Supplementary Figure S4 Network of co-cited reference (A) with corresponding clusters (B) and timeline view (C) for the 2017-2022 time period.

Supplementary Figure S5 Detail focus on all 13 extracted clusters of the co-citation reference networks ranked by burstness of citations for the time period 2017–2022.

Supplementary Figure S6 Network of co-cited reference (A) with corresponding clusters (B) and timeline view (C) for the year 2022.

Supplementary Figure S7 Overlay visualization of co-occurring authors’ keywords (A), and scored on the average publication year (B). Note: Minimum number of occurrences of a keyword= 10, 664 meet the thresholds, which are represented within 5 clusters. The nodes represent keywords and the colors show the average year of publication for each node. The size of a node is proportional to the frequency of keyword co-occurrence. The co-occurrence network is weighted on total link strength across different keyword node, and scored on the average publication years from 1989 to 2022.

Supplementary Figure S8 Annual scientific production (A) and average citation per year for references (B) (1989-2022).

Supplementary Figure S9 Top 10 growth source (1989–2022 and 2017–2022).

Supplementary Figure S10 Overlay visualization of most cited journals for the last 5 years (A), and most co-cited journals which published the most articles these last 30 years (B). Figure A is obtained with CiteSpace and Figure B with VOSviewer. (A) 13 clusters are identified. (B) Weighted on documents, Minimum number of citations of a journal= 150, 523 meet the thresholds, which are identified with 4 clusters.

Supplementary Figure S11 Co-authorship network obtained with VOSviewer. 51 clusters are identified comprising 625 different authors. Each cluster is identified with a different color. Minimum number of documents of an author= 10.

Supplementary Figure S12 Visualization of the author co-citation network (A), with corresponding clusters (B) and time map (C) (2017-2022).

Supplementary Table S1 Co-cited reference network detailed clusters (1989–2022, 2017–2022 and 2022)

Supplementary Table S2 Burstness analysis for countries, institutions, authors, references and keywords (1989–2022, 2017–2022 and 2022). Note: The beginning of a blue line depicts when an article is published. The beginning of a red segment marks the beginning of a period of burst, whereas the end of the red segment marks the end of the burst period.

Supplementary Table S3 Summary of the largest clusters identified for co-authorship network (1989–2022) obtained with CiteSpace.

Supplementary Table S4 Summary of the largest clusters identified for the author co-citation network (2017–2022) obtained with CiteSpace.

Supplementary Table S5 The top countries and institutions (1989–2022 and 2017–2022 period).

Supplementary Table S6 Average citations and total link strength of countries, institutions, journals, references, and authors per cluster based on bibliographic coupling analysis.
