# Supplementary figures and images for: Trends in worldwide research on cardiac fibrosis over the period 1989–2022: a bibliometric study
Source: Front Cardiovasc Med. 2023 Jun 5;10:1182606. doi: 10.3389/fcvm.2023.1182606 (PMC10277498; doi:10.3389/fcvm.2023.1182606)

TS (“topic,” including title, abstract, author’s keywords and keywords Plus)

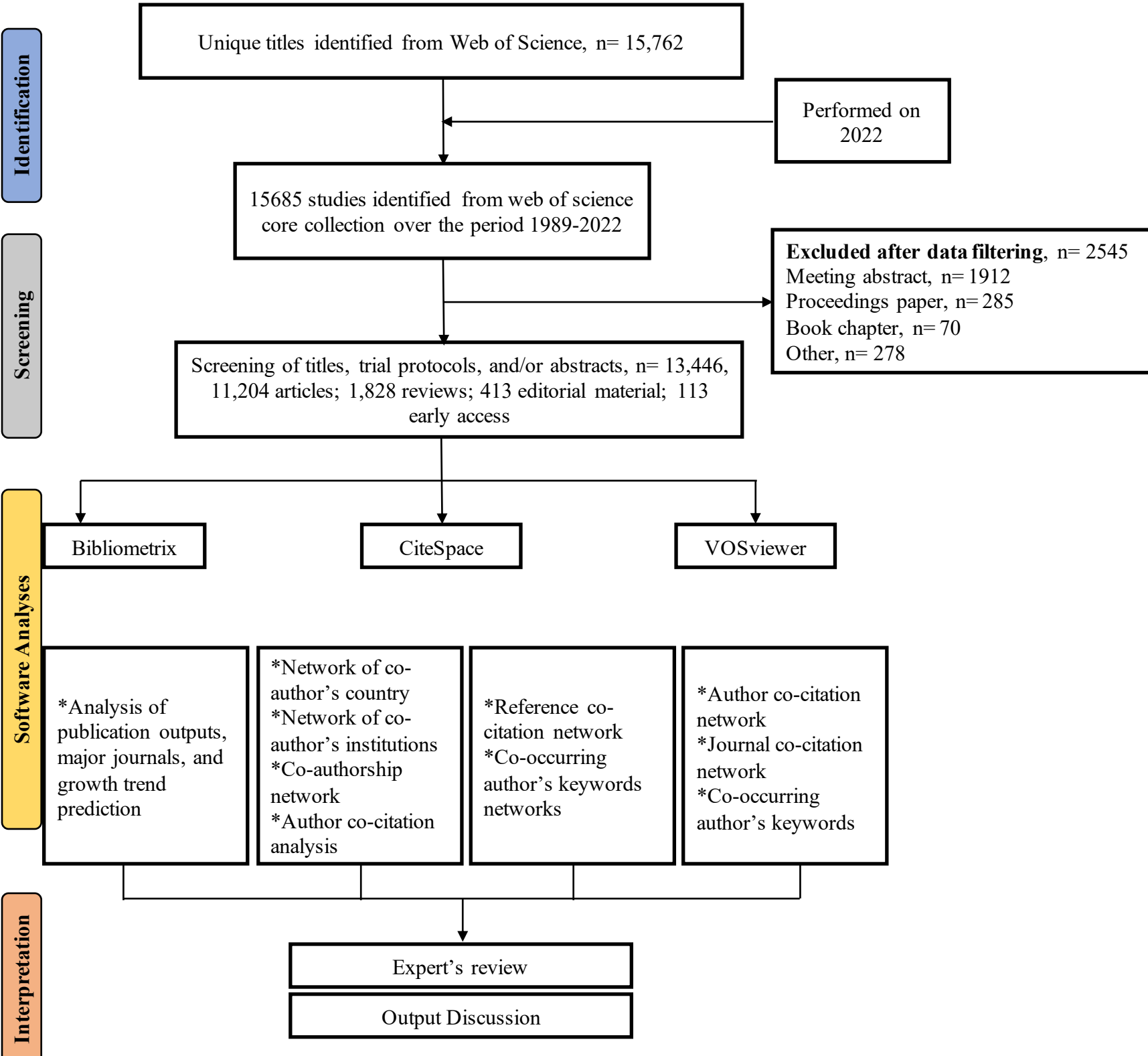

Supplement: Supplementary Figure S1 — Flow chart of the scientometric study. [file Image1.pdf]

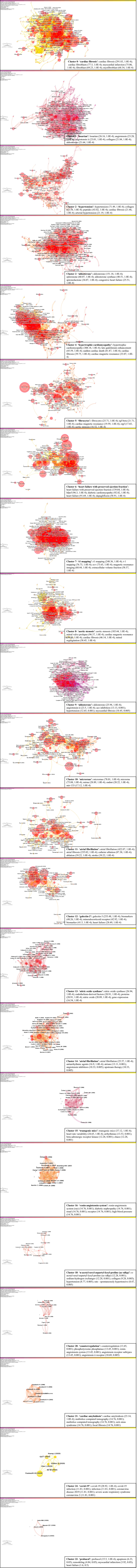

Supplement: Supplementary Figure S2 — Detail focus on most important clusters of the co-citation reference networks ranked by burstness of citations (1989-2022). For each cluster, we report all five top keywords obtained, the selected label being the keywords that are the most cited (generated by the likelihood ratio of keywords). These keywords are highly susceptible to represent the overall topic of a cluster. Burstness is represented in each cluster with red tree-rings around nodes. [file Image2.pdf]

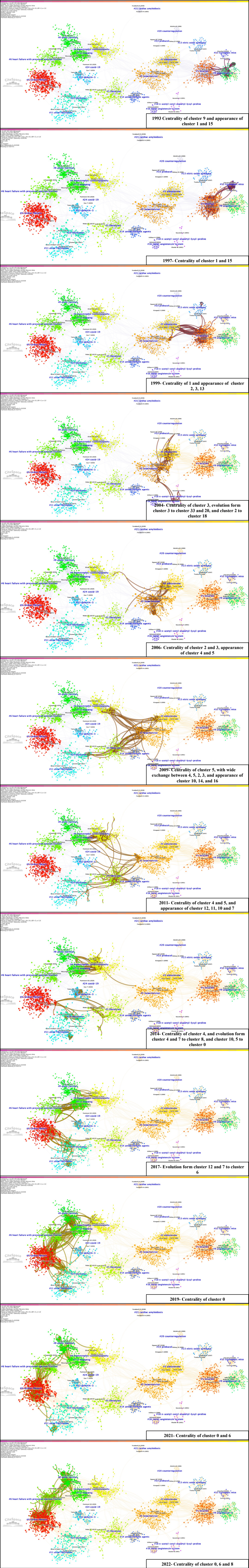

Supplement: Supplementary Figure S3 — Link walkthrough between clusters based on burstness dynamic for co-cited reference network (1989-2022). [file Image3.pdf]

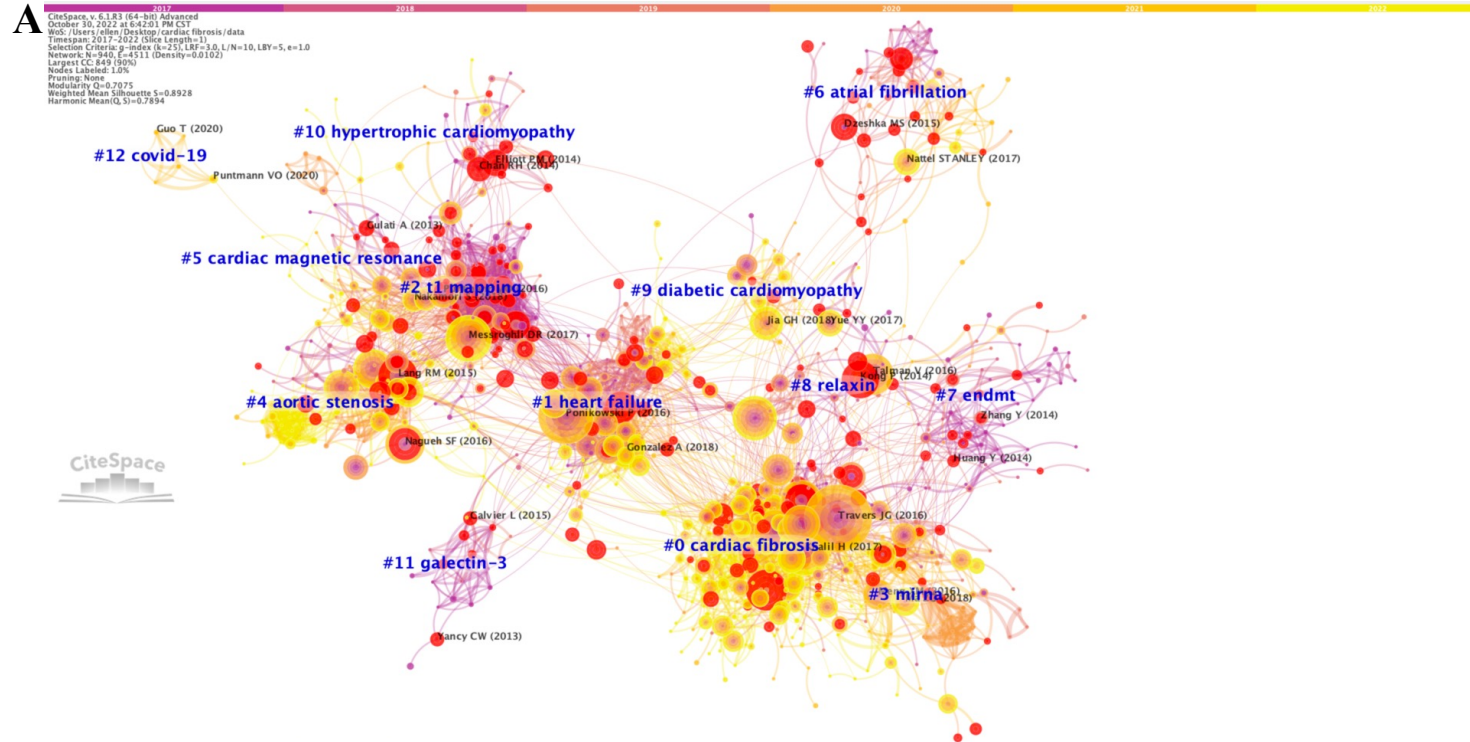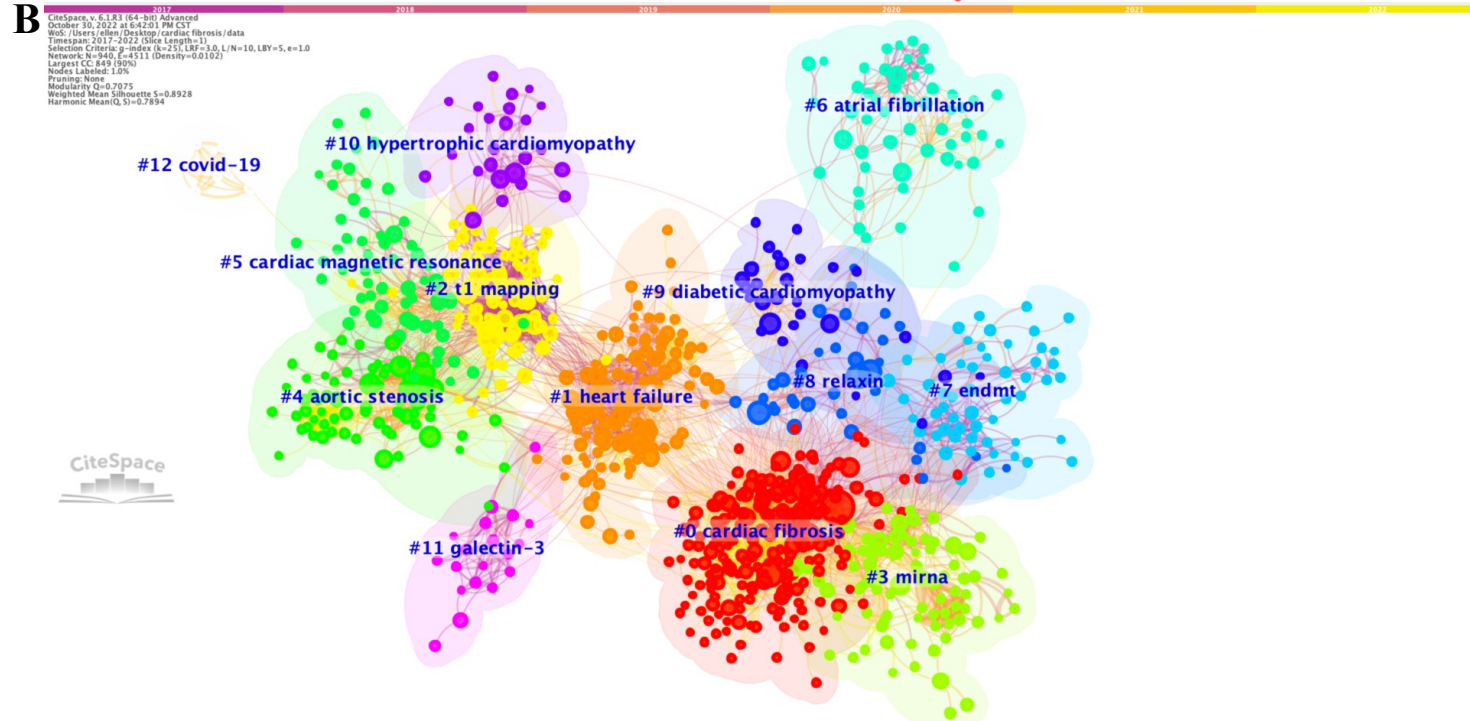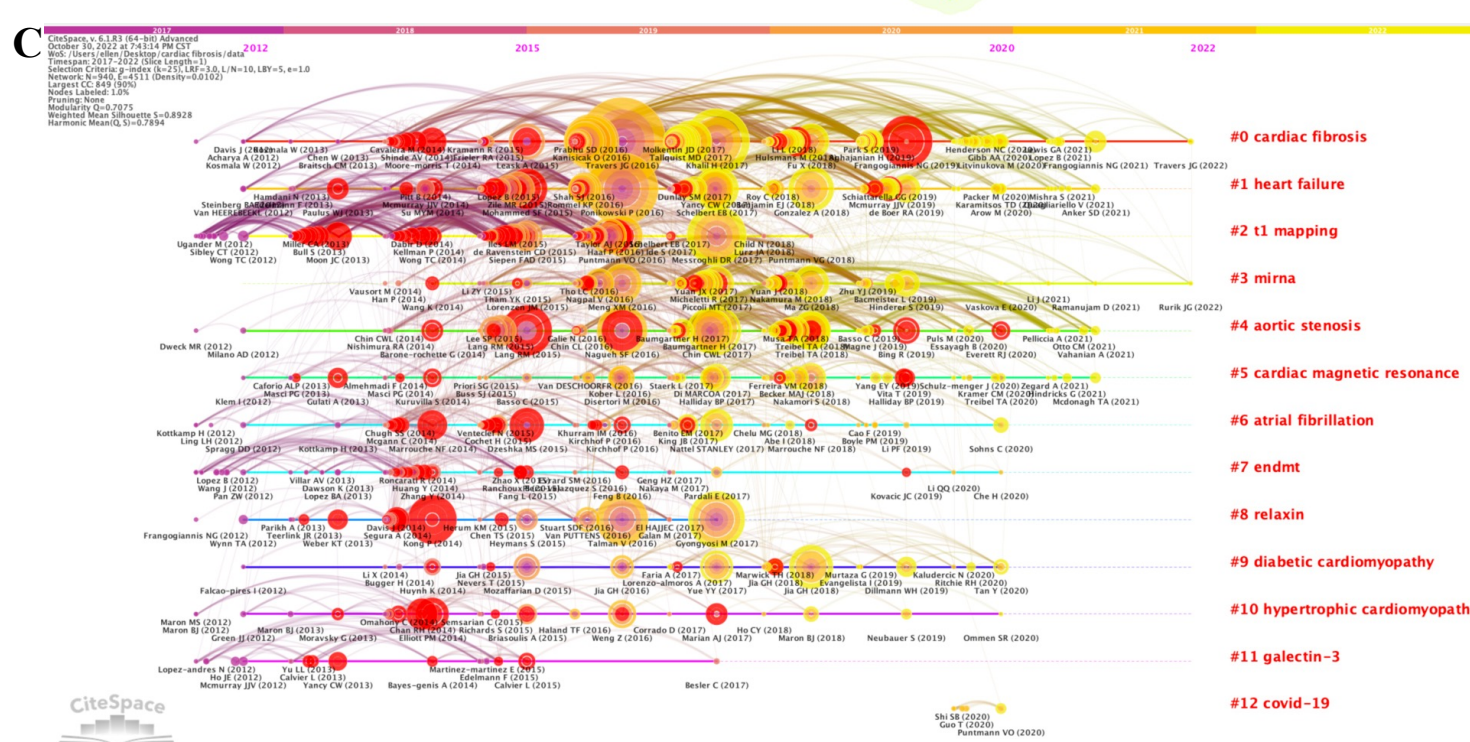

Supplement: Supplementary Figure S4 — Network of co-cited reference (A) with corresponding clusters (B) and timeline view (C) for the 2017-2022 time period. [file Image4.pdf]

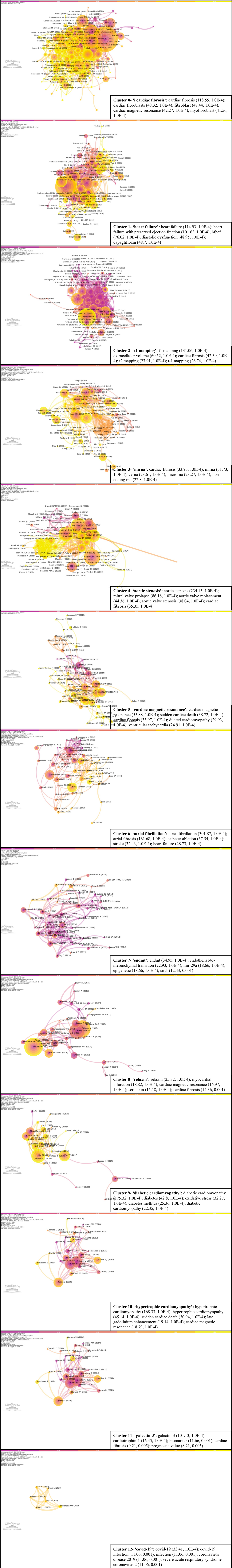

Supplement: Supplementary Figure S5 — Detail focus on all 13 extracted clusters of the co-citation reference networks ranked by burstness of citations for the time period 2017–2022. [file Image5.pdf]

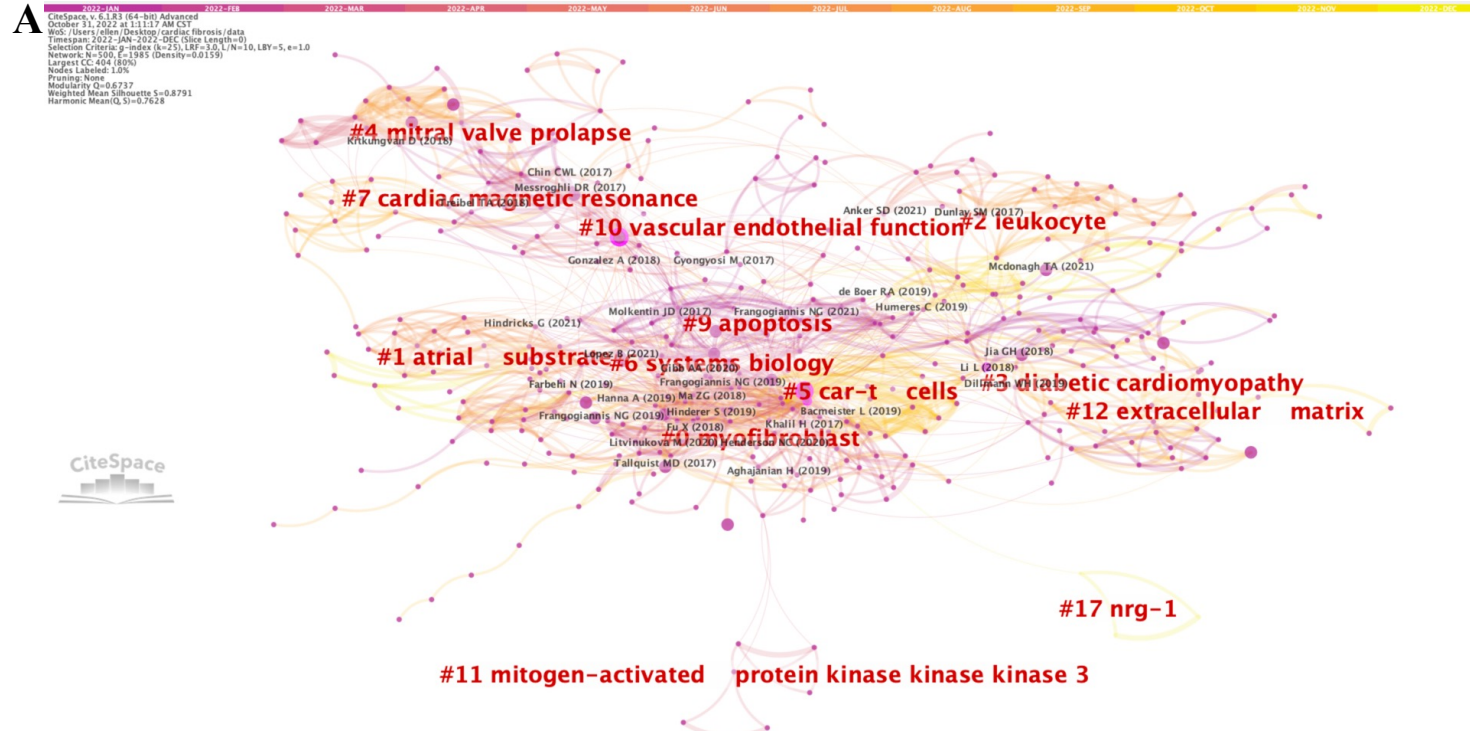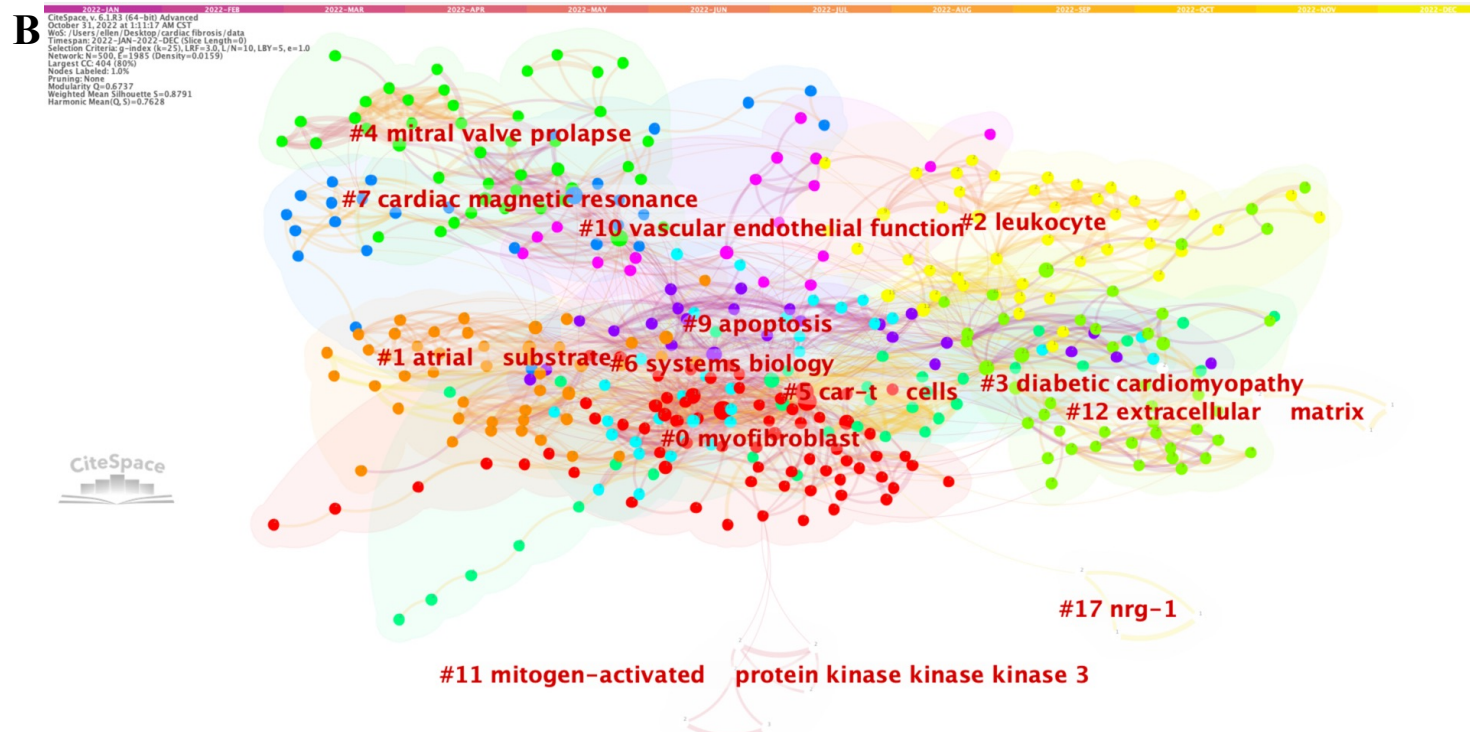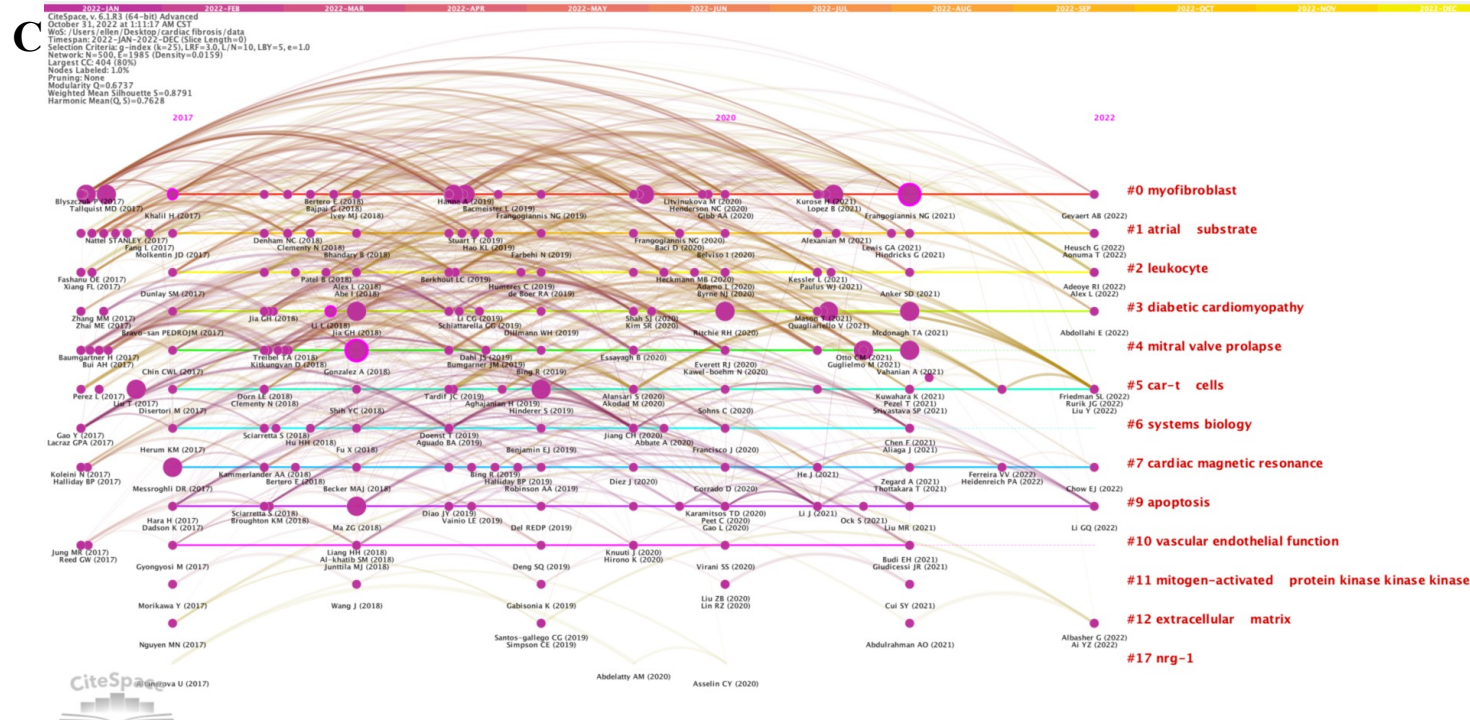

Supplement: Supplementary Figure S6 — Network of co-cited reference (A) with corresponding clusters (B) and timeline view (C) for the year 2022. [file Image6.pdf]

# B

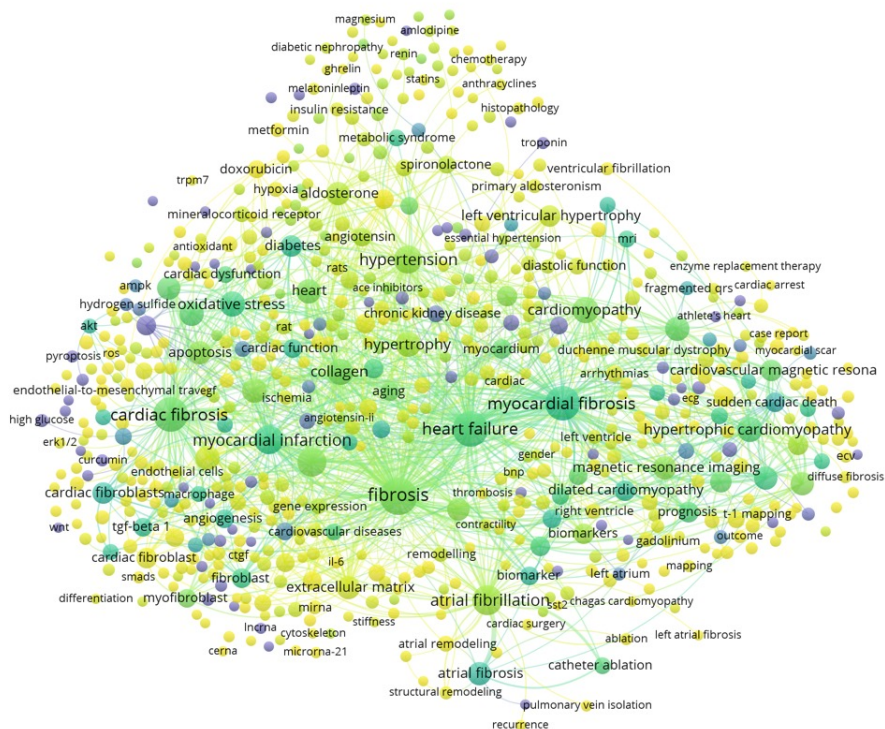

Supplement: Supplementary Figure S7 — Overlay visualization of co-occurring authors’ keywords (A), and scored on the average publication year (B). Note: Minimum number of occurrences of a keyword= 10, 664 meet the thresholds, which are represented within 5 clusters. The nodes represent keywords and the colors show the average year of publication for each node. The size of a node is proportional to the frequency of keyword co-occurrence. The co-occurrence network is weighted on total link strength across different keyword node, and scored on the average publication years from 1989 to 2022. [file Image7.pdf]

**A**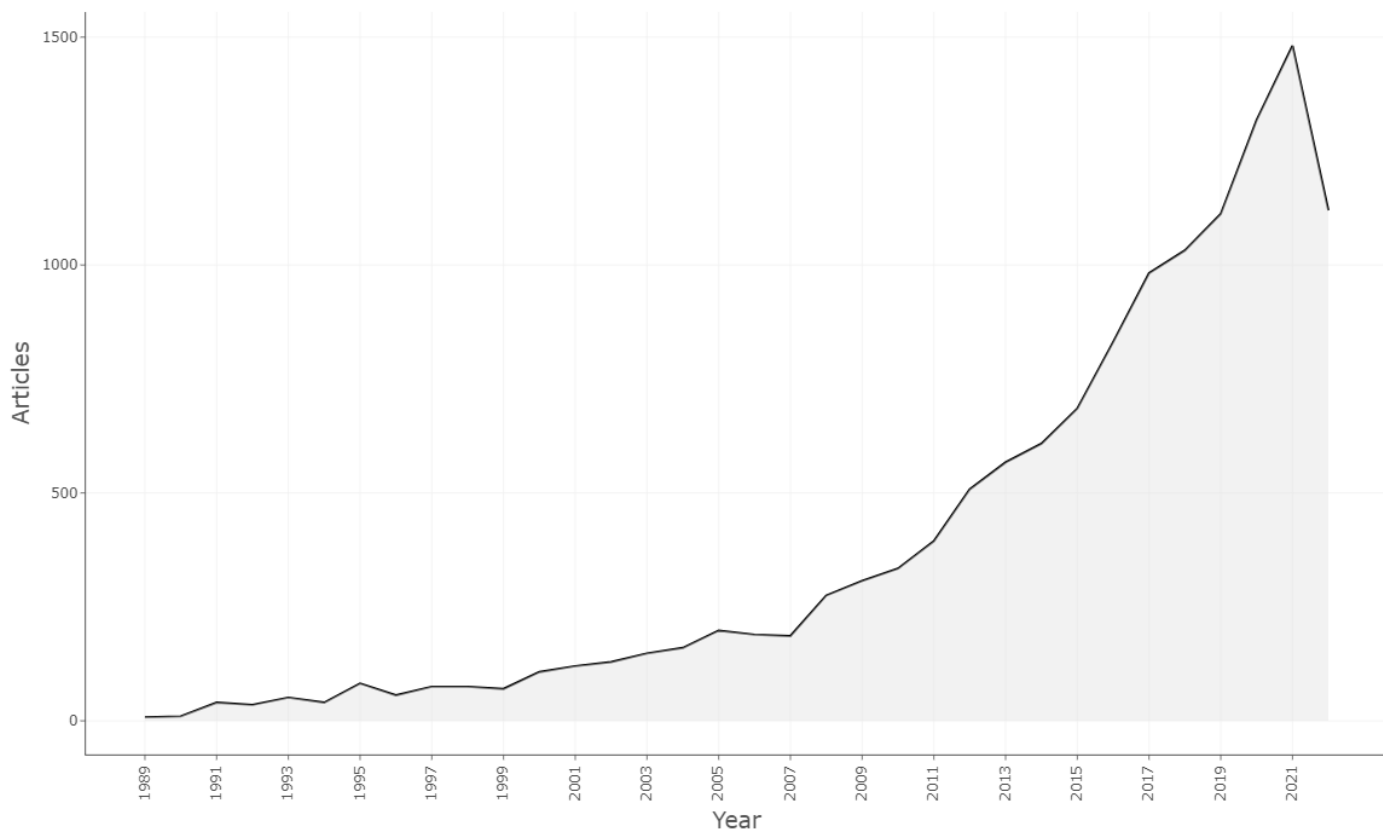**B**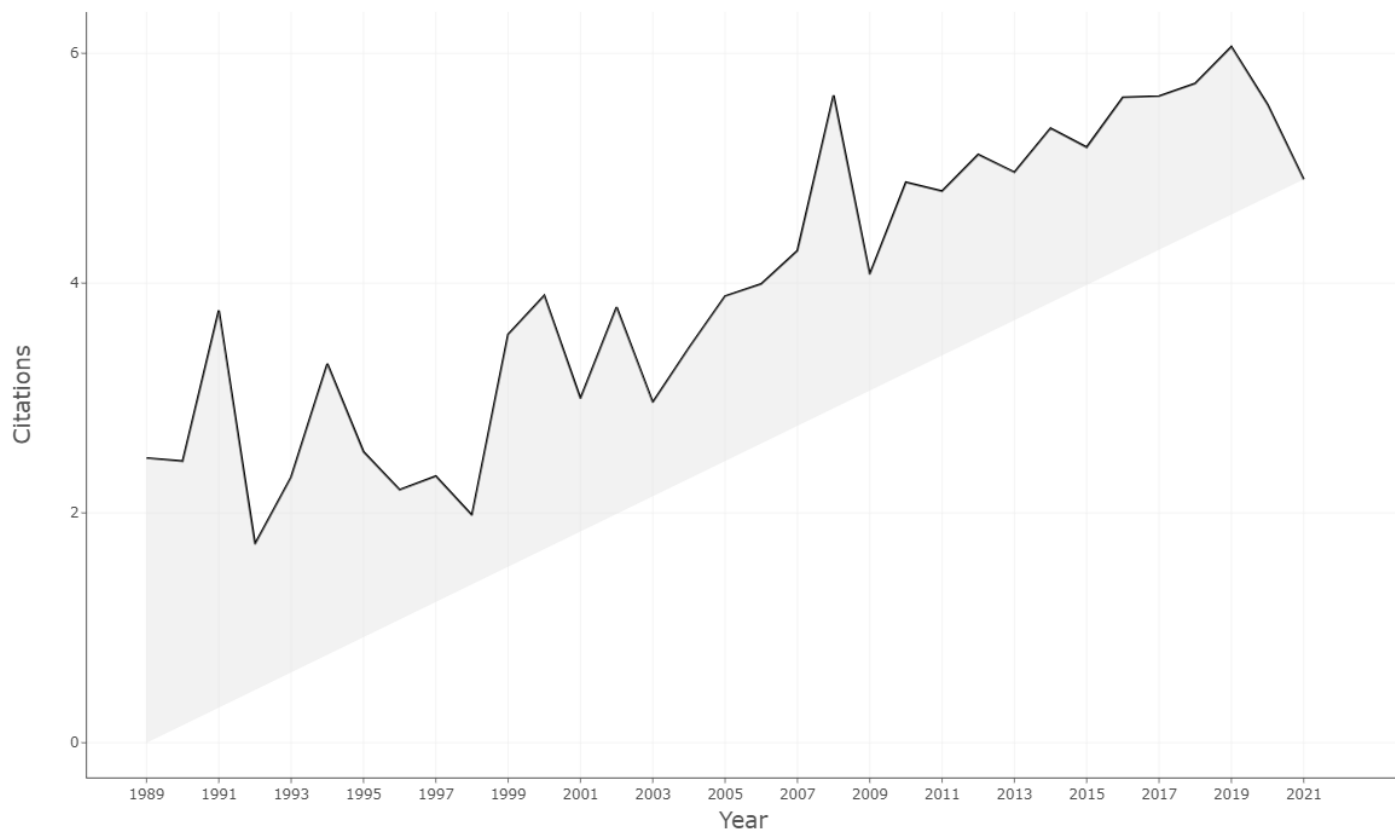

Supplement: Supplementary Figure S8 — Annual scientific production (A) and average citation per year for references (B) (1989-2022). [file Image8.pdf]

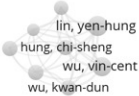

Supplement: Supplementary Figure S11 — Co-authorship network obtained with VOSviewer. 51 clusters are identified comprising 625 different authors. Each cluster is identified with a different color. Minimum number of documents of an author= 10. [file Image11.pdf]

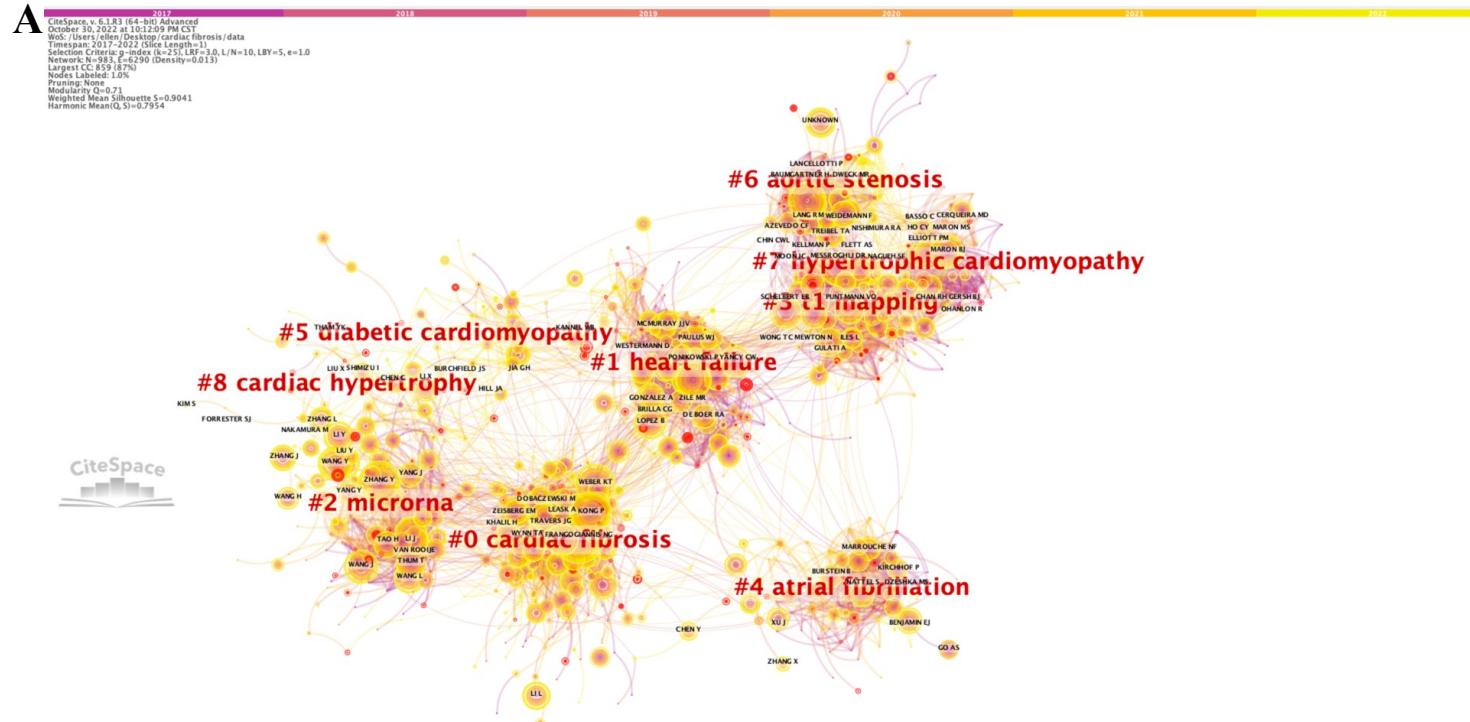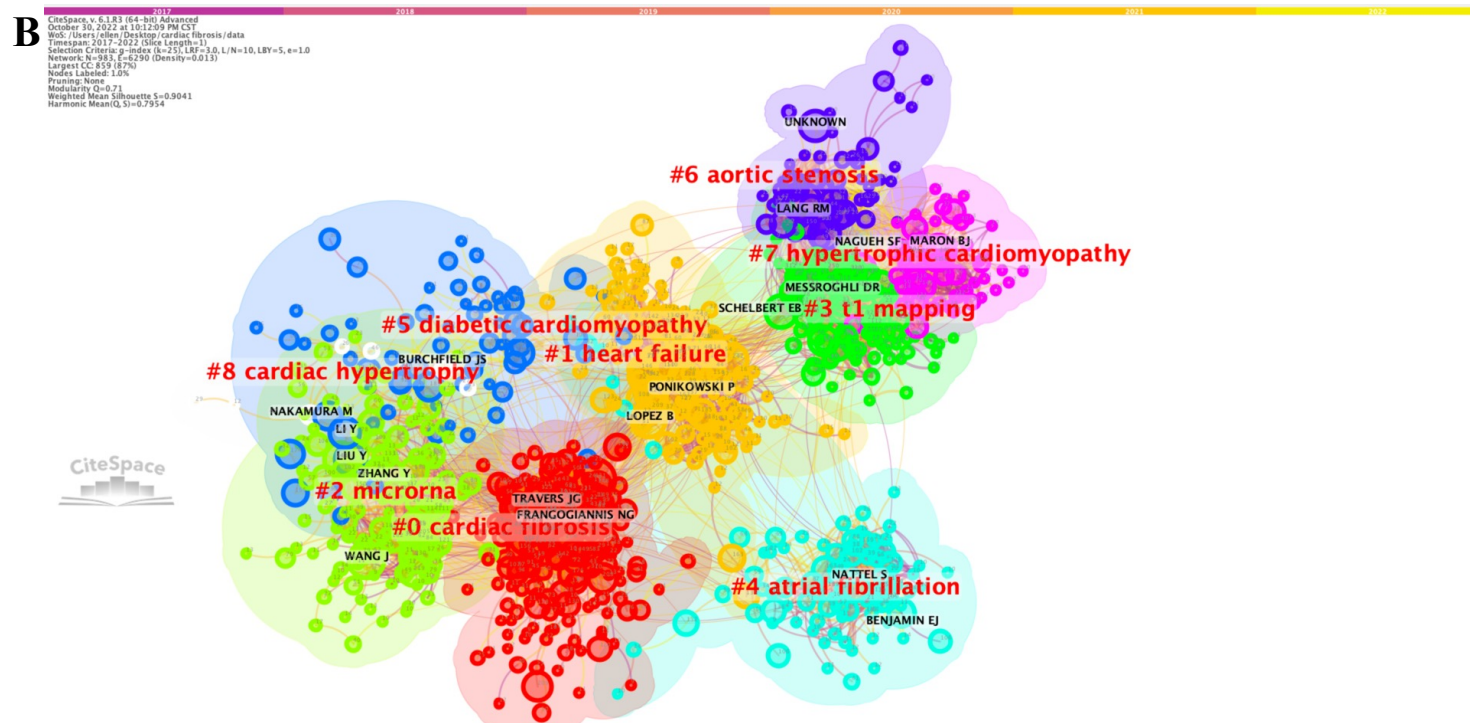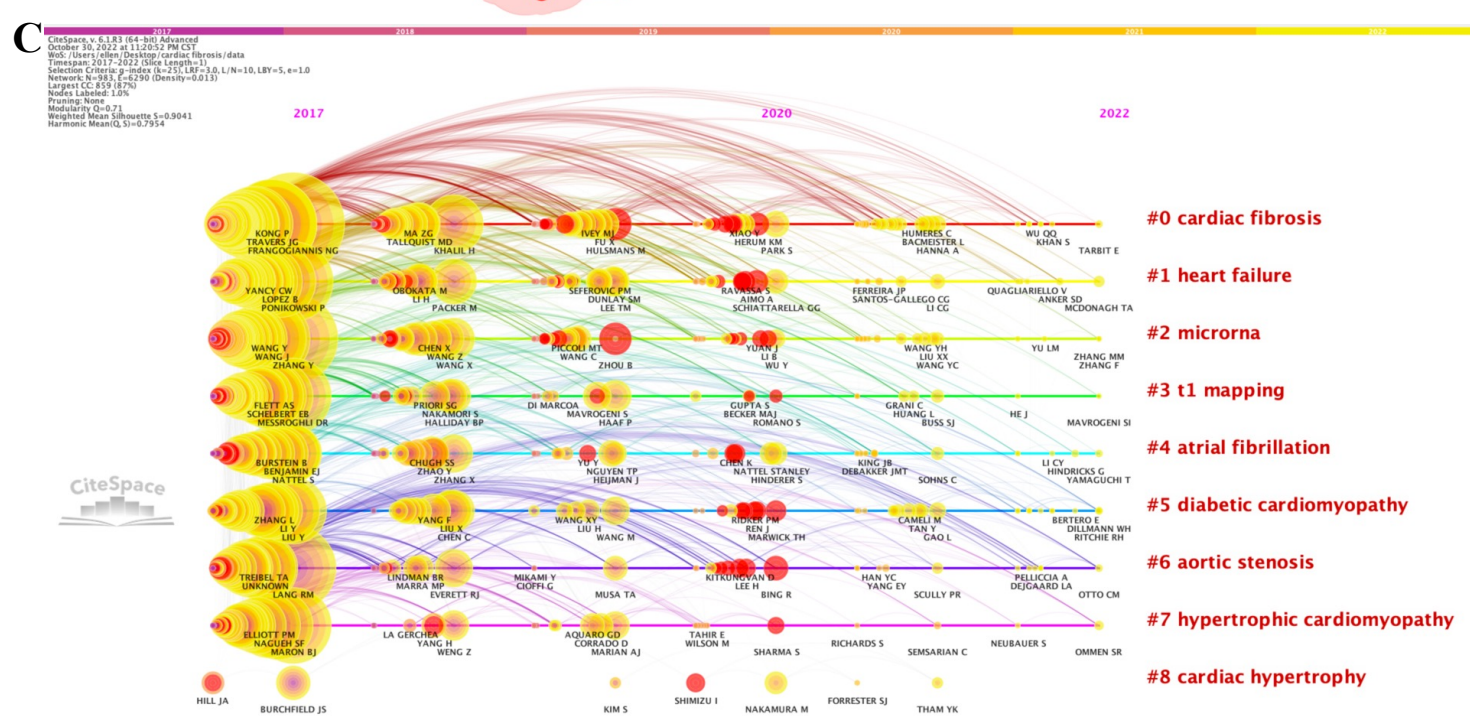

Supplement: Supplementary Figure S12 — Visualization of the author co-citation network (A), with corresponding clusters (B) and time map (C) (2017-2022). [file Image12.pdf]
