# Supplementary material for: Trends in worldwide research on cardiac fibrosis over the period 1989–2022: a bibliometric study
Source: Front Cardiovasc Med. 2023 Jun 5;10:1182606. doi: 10.3389/fcvm.2023.1182606 (PMC10277498; doi:10.3389/fcvm.2023.1182606)
Supplement: Supplementary Figure S9 — Top 10 growth source (1989–2022 and 2017–2022). [file Image9.pdf]

A

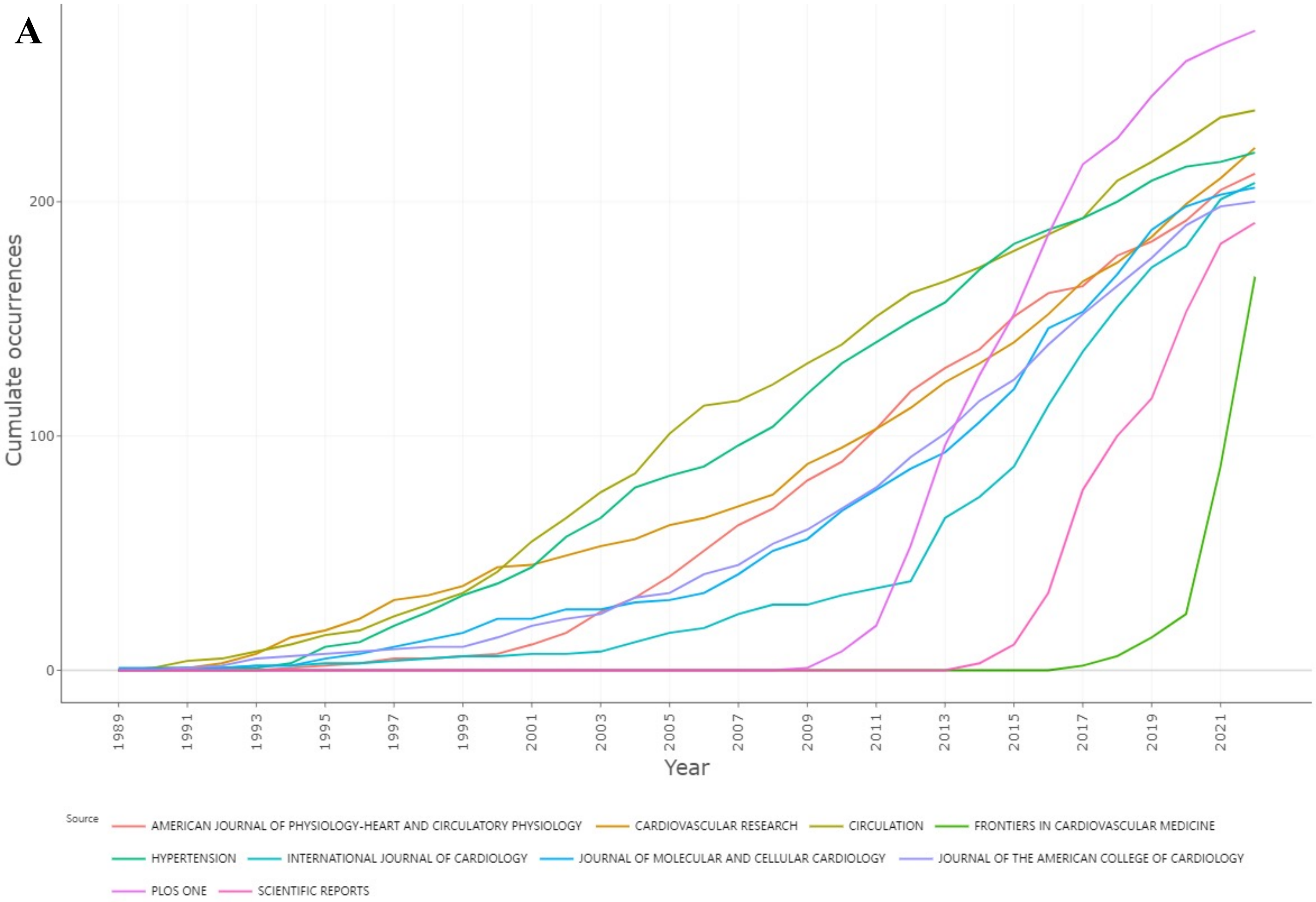
Cumulate occurrences
200
100
0
1989
1991
1993
1995
1997
1999
2001
2003
2005
2007
2009
2011
2013
2015
2017
2019
2021
Year
Source
AMERICAN JOURNAL OF PHYSIOLOGY-HEART AND CIRCULATORY PHYSIOLOGY
CARDIOVASCULAR RESEARCH
CIRCULATION
FRONTIERS IN CARDIOVASCULAR MEDICINE
HYPERTENSION
INTERNATIONAL JOURNAL OF CARDIOLOGY
JOURNAL OF MOLECULAR AND CELLULAR CARDIOLOGY
JOURNAL OF THE AMERICAN COLLEGE OF CARDIOLOGY
PLOS ONE
SCIENTIFIC REPORTS

B

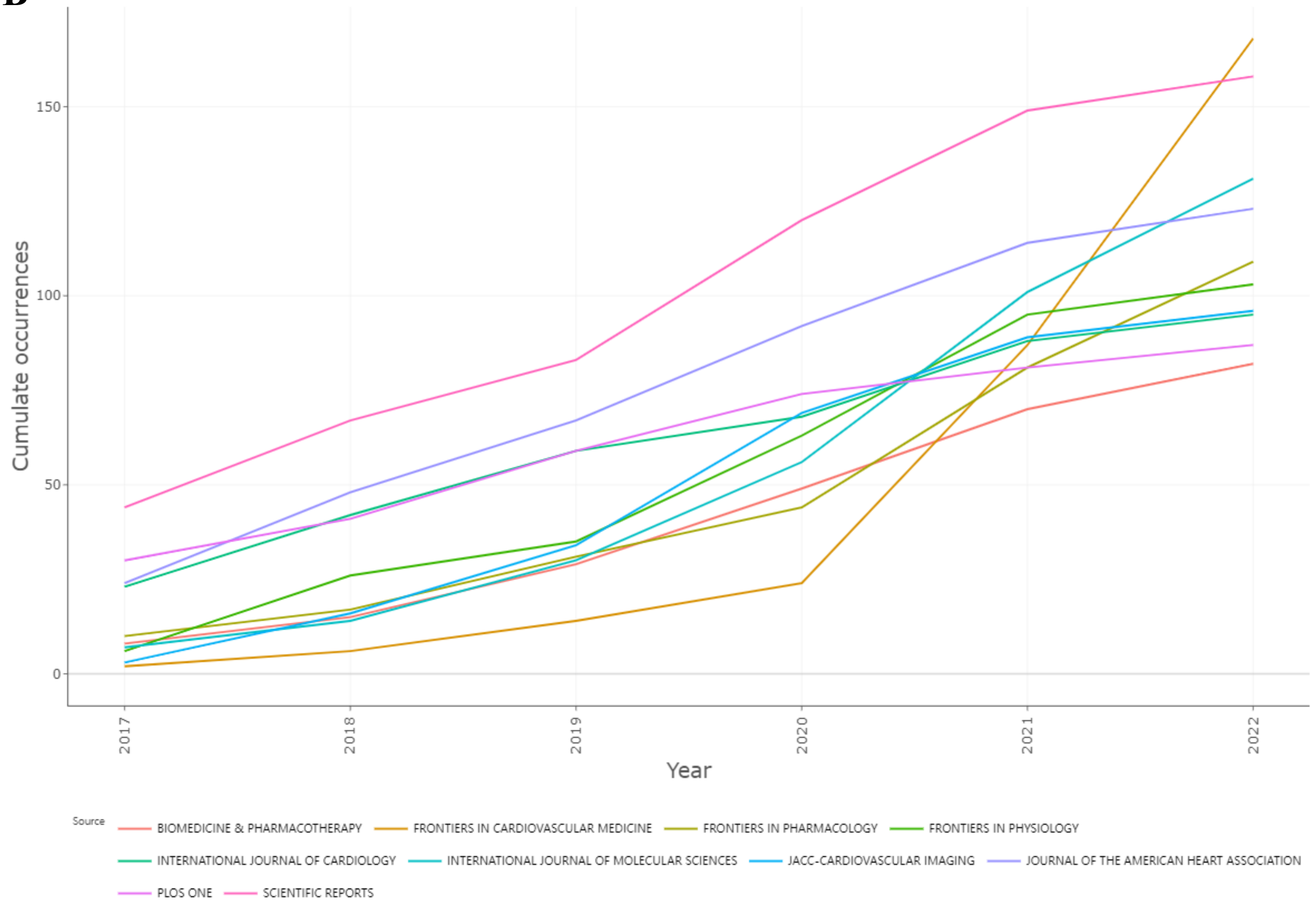
Cumulate occurrences
150
100
50
0
2017
2018
2019
2020
2021
2022
Year
Source
BIOMEDICINE & PHARMACOTHERAPY
FRONTIERS IN CARDIOVASCULAR MEDICINE
FRONTIERS IN PHARMACOLOGY
FRONTIERS IN PHYSIOLOGY
INTERNATIONAL JOURNAL OF CARDIOLOGY
INTERNATIONAL JOURNAL OF MOLECULAR SCIENCES
JACC-CARDIOVASCULAR IMAGING
JOURNAL OF THE AMERICAN HEART ASSOCIATION
PLOS ONE
SCIENTIFIC REPORTS
