# Supplementary material for: Trends in worldwide research on cardiac fibrosis over the period 1989–2022: a bibliometric study
Source: Front Cardiovasc Med. 2023 Jun 5;10:1182606. doi: 10.3389/fcvm.2023.1182606 (PMC10277498; doi:10.3389/fcvm.2023.1182606)
Supplement: Supplementary Figure S10 — Overlay visualization of most cited journals for the last 5 years (A), and most co-cited journals which published the most articles these last 30 years (B). Figure A is obtained with CiteSpace and Figure B with VOSviewer. (A) 13 clusters are identified. (B) Weighted on documents, Minimum number of citations of a journal= 150, 523 meet the thresholds, which are identified with 4 clusters. [file Image10.pdf]

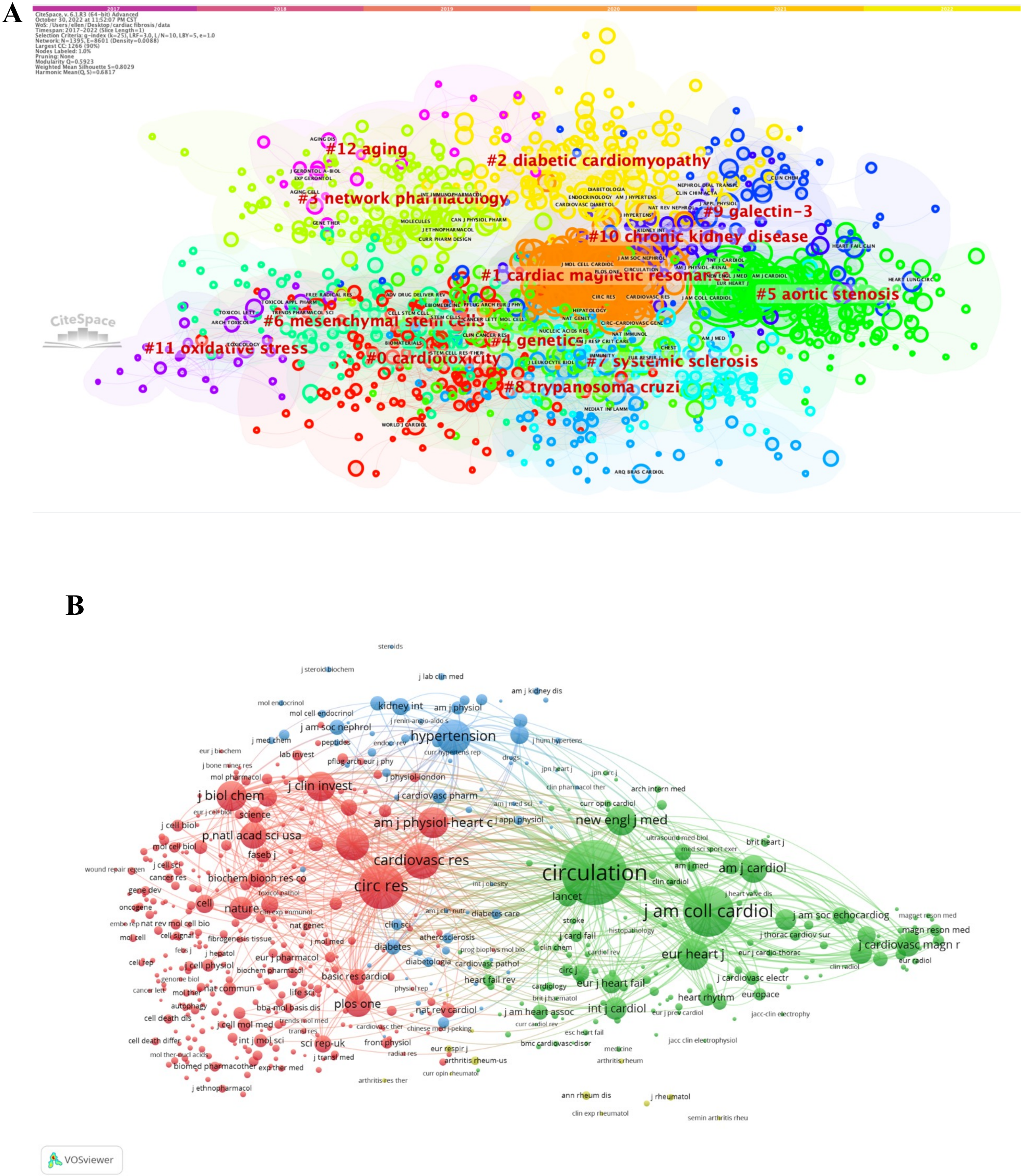
A
CiteSpace, v. 6.1.R3 (64-bit) Advanced
October 30, 2022 at 11:52:07 PM CST
WoS: /Users/ellen/Desktop/cardiac fibrosis/data
Timespan: 2017-2022 (Slice Length=1)
Selection Criteria: g-index (k=25), LRF=3.0, L/N=10, LBY=5, e=1.0
Network: N=1395, E=8601 (Density=0.0088)
Largest CC: 1266 (90%)
Nodes Labeled: 1.0%
Pruning: None
Modularity Q=0.5923
Weighted Mean Silhouette S=0.8029
Harmonic Mean(Q, S)=0.6817
2017
2018
2019
2020
2021
2022
#12 aging
#2 diabetic cardiomyopathy
#3 network pharmacology
#9 galectin-3
#10 chronic kidney disease
#1 cardiac magnetic resonance
#5 aortic stenosis
#6 mesenchymal stem cells
#11 oxidative stress
#4 genetic
#0 cardiotoxicity
#7 systemic sclerosis
#8 trypanosoma cruzi
CiteSpace
B
steroids
j steroid biochem
j lab clin med
am j kidney dis
mol endocrinol
mol cell endocrinol
kidney int
am j physiol
j am soc nephrol
hypertension
j med chem
peptides
j hum hypertens
eur j biochem
lab invest
pflug arch eur j phy
drugs
j physiol-london
j biol chem
j clin invest
j cardiovasc pharm
arch intern med
science
am j physiol-heart c
j appl physiol
new engl j med
j cell biol
p natl acad sci usa
faseb j
cardiovasc res
brit heart j
circulation
am j cardiol
biochem biophys res co
circ res
lancet
cell
nature
j am coll cardiol
j am soc echocardiog
diabetes care
magn reson med
clin sci
atherosclerosis
j card fail
j cardiovasc magn r
diabetes
eur heart j
eur j pharmacol
diabetologia
cardiovasc pathol
heart fail rev
basic res cardiol
eur j heart fail
j cardiovasc electr
nat commun
life sci
heart rhythm
europace
plos one
nat rev cardiol
j am heart assoc
int j cardiol
j cell mol med
int j mol sci
sci rep-uk
front physiol
eur respir j
arthritis rheum-us
biomed pharmacother
exp ther med
j ethnopharmacol
ann rheum dis
j rheumatol
clin exp rheumatol
semin arthritis rheu
VOSviewer
